# Comprehensive Mutation Analysis in Colorectal Flat Adenomas

**DOI:** 10.1371/journal.pone.0041963

**Published:** 2012-07-27

**Authors:** Quirinus J. M. Voorham, Beatriz Carvalho, Angela J. Spiertz, Bart Claes, Sandra Mongera, Nicole C. T. van Grieken, Heike Grabsch, Martin Kliment, Bjorn Rembacken, Mark A. van de Wiel, Philip Quirke, Chris J. J. Mulder, Diether Lambrechts, Manon van Engeland, Gerrit A. Meijer

**Affiliations:** 1 Department of Pathology, VU University Medical Center, Amsterdam, The Netherlands; 2 Department of Pathology, University Maastricht, Maastricht, The Netherlands; 3 The Vesalius Research Center, K.U. Leuven and VIB, Leuven, Belgium; 4 Vesalius Research Center, University of Leuven, Leuven, Belgium; 5 Pathology and Tumour Biology, Leeds Institute of Molecular Medicine, University of Leeds, Leeds, United Kingdom; 6 Gastroenterology, Hospital Vitkovice, Ostrava, Czech Republic; 7 Centre for Digestive Diseases, Leeds General Infirmary, Leeds, United Kingdom; 8 Department of Epidemiology and Biostatistics, VU University Medical Center, Amsterdam, The Netherlands; 9 Department of Gastroenterology, VU University Medical Center, Amsterdam, The Netherlands; IFOM, Fondazione Istituto FIRC di Oncologia Molecolare, Italy

## Abstract

**Background:**

Flat adenomas are a subgroup of colorectal adenomas that have been associated with a distinct biology and a more aggressive clinical behavior compared to their polypoid counterparts. In the present study, we aimed to compare the mutation spectrum of 14 cancer genes, between these two phenotypes.

**Methods:**

A consecutive series of 106 flat and 93 polypoid adenomas was analyzed retrospectively for frequently occurring mutations in “hot spot” regions of *KRAS*, *BRAF*, *PIK3CA* and *NRAS*, as well as selected mutations in *CTNNB1* (β-catenin), *EGFR*, *FBXW7* (*CDC4*), *PTEN*, *STK11*, *MAP2K4*, *SMAD4*, *PIK3R1* and *PDGFRA* using a high-throughput genotyping technique. Additionally, *APC* was analyzed using direct sequencing.

**Results:**

*APC* mutations were more frequent in polypoid adenomas compared to flat adenomas (48.5% versus 30.3%, respectively, p = 0.02). Mutations in *KRAS*, *BRAF*, *NRAS*, *FBXW7* and *CTNNB1* showed similar frequencies in both phenotypes. Between the different subtypes of flat adenomas (0-IIa, LST-F and LST-G) no differences were observed for any of the investigated genes.

**Conclusion:**

The lower *APC* mutation rate in flat adenomas compared to polypoid adenomas suggests that disruption of the Wnt-pathway may occur via different mechanisms in these two phenotypes. Furthermore, in contrast to previous observations our results in this large well-defined sample set indicate that there is no significant association between the different morphological phenotypes and mutations in key genes of the RAS-RAF-MAPK pathway.

## Introduction

It is now widely accepted that phenotypically different types of colorectal adenomas exist. Whereas the terms polyps and adenomas have long been used as synonyms, in 1985 Muto *et al* were the first to describe ‘small flat adenoma’ in the colorectum [1]. These flat adenomas have, until quite recently, been considered rare in Western countries, in contrast to Japan where they have been reported to represent 12–40% of colorectal adenomas or early carcinomas [2], [3]. However, due to advanced endoscopic imaging techniques and increasing awareness, similar incidence of flat adenomas are now reported in the West [4]–[6]. Flat lesions have been associated with a more aggressive behavior and are more likely to contain advanced histology [6], [7]. Flat adenomas therefore represent an important piece of the puzzle of colorectal cancer (CRC) pathogenesis.

CRC results from the accumulation of multiple genetic and epigenetic alterations in the epithelial cells that line the large intestine. These events first give rise to an adenoma that, in a minority of cases progresses into an invasive, metastasizing adenocarcinoma [8]. These genetic and epigenetic alterations affect different pathways that regulate multiple biological processes critical to cancer development [9].

One of these is the Wnt-signaling pathway, and somatic mutations in the tumor suppressor gene, *APC*, a key-player in the Wnt-pathway, are common in CRC [10]. Wild type APC forms a complex with other proteins to coordinate β-catenin degradation. When *APC* is mutated, degradation of β-catenin fails, resulting in translocation of β-catenin to the nucleus where it initiates transcription of key proliferation genes. We previously found significantly more chromosome 5q loss (*APC* locus) in flat adenomas compared to polypoid adenomas [11]. Next to disruption of *APC*, mutations in *CTNNB1* (β-catenin) are a (less common) way to constitutively activate the Wnt-signaling pathway [12].

Another key pathway in CRC tumorigenesis is the RAS-RAF-MAPK pathway, which is known to be one of the major pathways in the pathogenesis of CRC. Disruption of this pathway at multiple levels occurs in many cancer types. Interestingly, initial molecular studies indicated a lower incidence of *KRAS* mutations in flat lesions compared to polypoid lesions [13], [14], while more recent studies contradict these findings [15], [16]. Similar contradicting results have been described for other mutated genes, including *BRAF* [16], [17].

Most studies investigating flat adenomas, however, have been performed in relatively small and selected sample series, which may cause these conflicting findings. To further elucidate the role of the RAS-RAF-MAPK pathway in colorectal adenomas it is necessary to determine mutation frequencies of RAS-RAF-MAPK pathway genes, including upstream regulators and downstream effectors. For many other important cancer related genes, even less data on mutation status in flat adenomas exist, and consequently, much of the genomics of flat colorectal adenomas remains to be elucidated.

The aim of the present study was to address this issue in a more systematic fashion, by analyzing the mutation spectrum of 14 well-known cancer genes (i.e. *BRAF*, *NRAS*, *KRAS*, *PIK3CA*, *PIK3R1*, *EGFR*, *PTEN*, *MAP2K4*, *SMAD4*, *FBXW7* (*CDC4*), *CTNNB1* (β-catenin), *STK11*, *PDGFRA* and *APC*) [18] in a large well-defined series of flat and polypoid adenomas using a high-throughput genotyping technique.

## Methods

### Ethical Statement

Collection, storage and use of archival tissue and patient data were performed in compliance with the “Code for Proper Secondary Use of Human Tissue in the Netherlands” (http://www.fmwv.nl and www.federa.org). This study was approved by the VU University medical center (2011–03), the Leeds University (CA02/014 Leeds (West)) and the Hospital Vitkovice (EK/140/10).

This study followed the ethical guidelines of the Institutional Review Board (IRB). The IRB waived the need for consent for use of the archive samples, and the samples were analyzed anonymously.

### Patient and Sample Selection

Formaldehyde-fixed, paraffin-embedded (FFPE) tissue samples from both a consecutive series of polypoid adenomas and a consecutive series of flat colorectal adenomas were collected at three different institutes. Excluding criteria were hereditary CRC, inflammatory bowel disease (IBD), all hyperplastic polyps as well as all adenomas smaller than 5 mm (except for two depressed adenomas, subtype IIc, since these were of clinical interest and unequivocally non-polypoid), resulting in a series of 106 flat adenomas and 93 polypoid adenomas. Selective chromo-endoscopy (dye-spraying), which has been suggested to enhance the detection rate of flat lesions [19] was used when applicable. Of these 199 samples, 55 adenomas originated from 53 patients from the Leeds General Infirmary, Leeds, UK, collected between 1995–2007 [5], 51 adenomas originated from 47 patients from the Hospital Vitkovice, Ostrava, Czech Republic, collected between 2006–2008 [20] and 93 adenomas originated from 76 patients from the VU University medical center, Amsterdam, The Netherlands, collected between 1990–2008. Endoscopically, flat adenomas were defined as height <2–3 mm. Classification of the adenomas was performed according to the Paris classification [21]. Five categories of adenomas were distinguished; polypoid/pedunculated type, 0-Ip (n = 93), slightly elevated type, 0-IIa (n = 40), slightly depressed type, 0-IIc (n = 4), lateral spreading type flat, LST-F (n = 41) and lateral spreading type granular, LST-G (n = 21), with LST being defined as a subclass of type 0-IIa measuring more than 10 mm in maximum diameter [22]. A summary of all clinical characteristics is listed in Table 1.

**Table 1:** Clinical characteristics of the 199 investigated adenomas.

	Polypoid Adenomas	Flat Adenomas
Total	n = 93 (%)	n = 106 (%)
Histology		
Tubular	46 (49)	73 (69)
Tubulovillous	36 (40)	27 (25)
Villous	10 (11)	2 (2)
Serrated (SSL)	0 (0)	3 (3)
Serrated (TSA)	0 (0)	1 (1)
Dysplasia		
LGD (mild)	11 (12)	10 (9)
LGD (moderate)	69 (74)	86 (81)
HGD (severe)	13 (14)	10 (9)
Size (mm)	17.0 [5–64]	16.2 [3–100]
Male/Female	41/52	66/40
Location		
Proximal	22 (24)	69 (65)
Distal	34 (37)	19 (18)
Rectum	33 (35)	16 (15)
Unknown	4 (4)	2 (2)
Age (years)	68.9 [40–90]	68.0 [45–87]
Paris classification		
0-Ip	93 (100)	
0-IIa		40 (38)
0-IIc		4 (4)
LST-G		21 (15)
LST-F		41 (39)

All adenomas were classified endoscopically using the Paris classification; SSL; sessile serrated lesion, TSA; traditional serrated adenoma, LGD, low grade dysplasia; HGD, high grade dysplasia; proximal colon includes caecum, ascending and transverse colon; distal colon includes sigmoid, descending and splenic flexure.

### DNA Isolation

DNA from FFPE material was isolated following macro-dissection (>70% dysplastic cells) as described before [23] with a few modifications. In stead of one day, a five day incubation period with lysis buffer (ATL buffer, QIAmp, DNA micro-kit, Qiagen, Venlo, The Netherlands) and freshly added (once every day) proteinase K (10 µl of 20 ng/µl) was performed. DNA was isolated using a column based method (QIAmp, DNA micro-kit, Qiagen, Venlo, The Netherlands). DNA concentrations and purities were measured on a Nanodrop ND-1000 spectrophotometer (Isogen, IJsselstein, The Netherlands).

### Mutation Analysis

**Figure 1:**
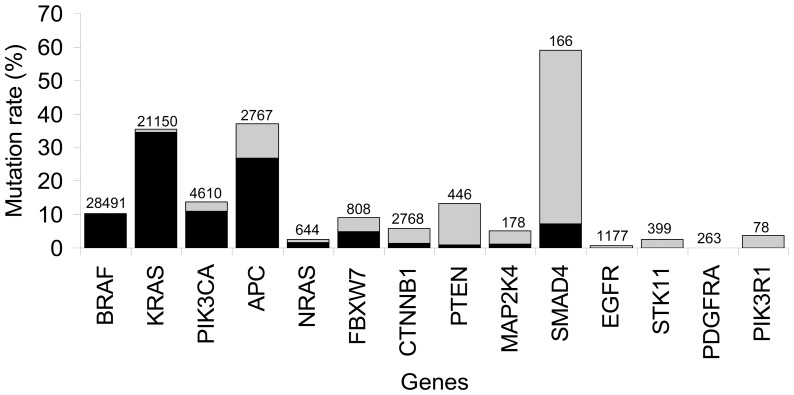
Total coverage of the 14 genes investigated, based on the COSMIC database. On the x-axis the analyzed gene. On the y-axis the mutation percentage is shown, based on large intestine carcinomas in the COSMIC database. In grey, the mutation percentages according to the COSMIC database, in black the mutation percentage covered by this study, on top of the bars the total number of analyzed samples found in the COSMIC database.

The Catalogue of Somatic Mutations in Cancer (COSMIC) database [24] was queried for mutations as described before [25]. Briefly, the most frequent mutations occurring in large intestinal cancer for *KRAS*, *BRAF*, *NRAS* and *PIK3CA* were selected. This resulted in a coverage of 97.9%, 97.3%, 81.4% and 68.8% for respectively *BRAF*, *KRAS*, *PIK3CA* and *NRAS* mutations (Figure 1, Table S1). For other genes of interest, no large series of CRC had been profiled or no specific mutation hot-spot could be identified. Therefore, for these genes, i.e. *CTNNB1*, *EGFR*, *FBXW7*, *PTEN*, *STK11*, *MAP2K4*, *NRAS*, *SMAD4*, *PIK3R1*, and *PDGFRA*, the COSMIC database was queried for the nucleotides most frequently mutated in all cancer types present in the database (Figure 1, Table S1).

Samples were genotyped for the respective nucleotide positions using a high-throughput matrix-assisted laser desorption/ionization–time of flight (MALDI-TOF) mass spectrometer (SpectroREADER, Sequenom, San Diego, CA, USA), at the Vesalius Research Center (Leuven, Belgium) as described previously [25], [26]. The sensitivity and accuracy of this technique have been documented before [25], [26]. Samples were considered of sufficient quality when more than 75% of the investigated nucleotide positions were reliably genotyped.

### APC Mutation Analysis

Mutations in the mutation cluster region (MCR1286-1513) of *APC* were analyzed by sequencing between codons 1260 and 1530 by four flanking PCRs followed by two semi-nested PCRs as described previously [27], using a 3500 Genetic Analyzer (Applied Biosystems Foster city, CA, USA). VectorNTI (Invitrogen) and Mutation Surveyor (SoftGenetics) were used for data analysis. Mutations were confirmed by independent PCR reactions and sequencing.

For 39 adenomas *APC* mutation status was determined and published previously [28]. Briefly, *APC* was analyzed using four flanking PCRs for the region 1291–1541 and sequenced on an ALFExpress (Amersham Biosciences Europe GmbH, Roosendaal, The Netherlands) DNA sequencer. Data was analyzed using the ALFwin sequence analyzer 2.10 program (Amersham Biosciences Europe GmbH).

### Microsatellite Instability (MSI) Status

MSI analysis was performed using the MSI Analysis System (MSI Multiplex System Version 1.2, Promega, Madison, WI, USA) consisting of five quasi-monomorphic mononucleotide markers (BAT-25, BAT-26, NR-21, NR-24, MONO-27) according to the manufacturer’s instructions. PCR products were separated using a 3130 Genetic Analyzer (Applied Biosystems, Foster City, CA, USA), and analyzed using GeneScan3100 (Applied Biosystems). An internal lane size standard was added to the PCR samples for accurate sizing of alleles and to adjust for run-to-run variations. When two or more markers were instable, the sample was interpreted as microsatellite instable (MSI), all other samples were classified as microsatellite stable (MSS).

### Data Analyses

To deal with partly missing data for samples with one or more unsuccessful assays, random imputation was used. This avoids underestimation (respectively overestimation) of the mutation frequency for one single gene that would be caused by discarding samples that had one or more unsuccessful assays, but showed evidence (respectively *no* evidence) of mutations for the successful assays. More specifically, each missing value was imputed to be a mutation with probability equal to the proportion of mutations at that specific nucleotide position, as computed from the non-missing data. Multiple random imputations were applied to obtain a more precise estimate of mutation percentage. Finally, odds ratios and differences thereof were estimated in order to compare the two groups. Using the imputed data, differences in frequencies of mutations between the different lesion types were evaluated by chi-square or Fisher’s exact test where appropriate. The results were pooled over the multiple random imputations, thereby accounting for the additional noise introduced by the imputation process.

Statistical language R, version 2.9.0 was used for the imputation and SPSS version 15.0 was used to perform the chi-square or Fisher’s exact test. All reported p-values are two-sided, and a p-value <0.05 was considered statistically significant.

## Results

In total 93 polypoid adenomas and 106 flat adenomas were analyzed for 13 genes (*BRAF*, *NRAS*, *KRAS*, *PIK3CA*, *PIK3R1*, *EGFR*, *PTEN*, *MAP2K4*, *SMAD4*, *FBXW7*, *CTNNB1*, *STK11* and *PDGFRA*) at 82 nucleotide positions. After excluding unreliable nucleotide positions (genotyped in less then 75% of all samples), 75 positions were left to be investigated. No mutations were found for eight genes, i.e. *PIK3CA, PIK3R1*, *EGFR*, *PTEN*, *MAP2K4*, *SMAD4*, *STK11* and *PDGFRA* (Table S2) leaving five genes, analyzed at 23 nucleotide positions, that contained mutations (*BRAF*, *NRAS*, *KRAS*, *FBXW7*, *CTNNB1*). For *APC*, the MCR was successfully sequenced and analyzed for 155 adenomas, i.e. 81 polypoid adenomas and 74 flat adenomas. (Figure 1, Table S1 and S2).

### KRAS

For *KRAS*, eight out of the nine nucleotide positions tested showed sufficient results (codon 12: c.34G>CAT, c.35G>ATC, codon 13: c.38G>ACT, c.39C>AGT, codon 59: c.175G>A, codon 61: c.181C>AG, c.182A>TGC, c.183A>CT) covering 98.85% of the mutations described in colorectal adenomas (Figure 1, Table S1C). Both flat and polypoid adenomas were most frequently mutated at codon 12, position 35 (60% (17/28) and 59% (20/33) for flat and polypoid adenomas, respectively) and transition from G>A was the most common nucleotide shift for both phenotypes (53% for flat adenomas and 50% for polypoid adenomas) (Table S2C and S3). The mutation at position c.182A>T (p.Q61L), was found in only one flat adenoma and has not been described before in colorectal adenomas. The nucleotide positions 39, 175, 181 and 183 showed no mutations (Table S2C). A significant difference between flat adenomas and polypoid adenomas was found at codon 12, position 34 (10.7% in polypoid adenomas versus 2.9% in flat adenomas, p = 0.05) and for codon 12, position 34 and 35 together (32.6% versus 20.0%, respectively, p = 0.05).

**Figure 2:**
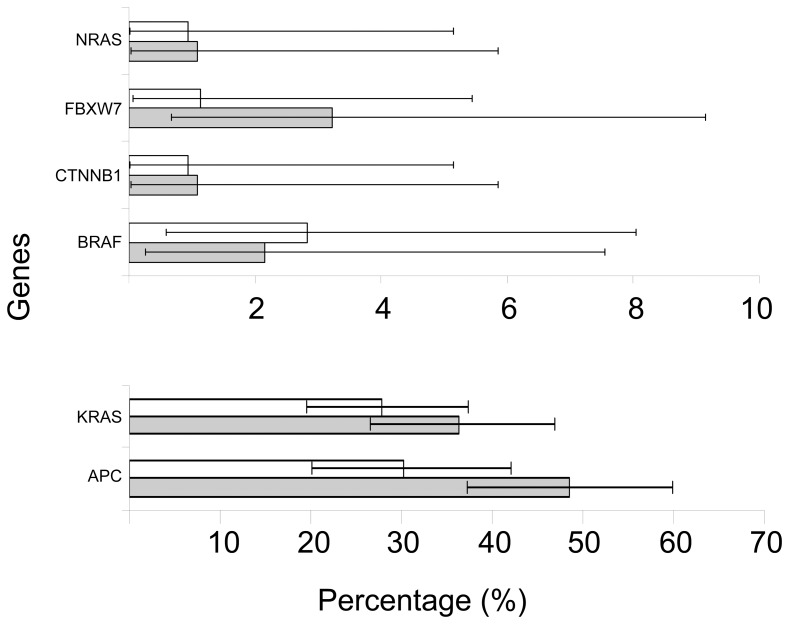
The mutation percentage for the flat and polypoid adenomas. Only genes that show a mutation in one of the adenoma types are included. White bars represent flat adenomas and grey bars polypoid adenomas. Percentages are calculated using random imputation, horizontal bars represent the 95% confidence interval.

Overall, *KRAS* mutations were observed in 36.30% (95% confidence interval (CI) 26.57%-46.92%) of polypoid adenomas and 27.85% (95% CI 19.58%-37.39%) of flat adenomas (Figure 2, Table S2C), resulting in no statistically significant difference (p = 0.2, odds ratio 0.67 (95% CI 0.37%-1.24%)).

### Relationship between KRAS Mutation and Adenoma Location

In the present series, flat adenomas were more commonly located proximal than polypoid adenomas (65% versus 24%, respectively, p = 7.7*10^−9^). No significant difference in overall *KRAS* mutation frequency was found after stratifying the adenomas by location (data not shown).

### Relationship between KRAS Mutation and Subtypes of Flat Adenomas

By analyzing the different subtypes of flat adenomas separately, no relationship was found between *KRAS* mutation and any of the subtypes. None of the four type IIc lesions, showed a *KRAS* mutation. The other three flat adenoma subtypes showed overall *KRAS* mutation rates of 26.8% (95% CI 14.22 to 42.94%), 26.73% (95% CI 14.16%-42.82%) and 34.67% (95% CI 15.58%-58.26%) for IIa, LST-F and LST-G, respectively. These *KRAS* mutation frequencies were not significantly different compared to *KRAS* mutation frequencies of polypoid adenomas (data not shown).

### BRAF

Two of the four nucleotide positions known to carry *BRAF* mutations in CRC were successfully analyzed, including c.1799T>A (p.V600E), (with 88.64% the most frequently mutated *BRAF* position in adenomas, Figure 1, Table S1A). In the present study, the *BRAF* mutation frequency was 2.83% (95% CI 0.59%-8.05%) in flat adenomas and 2.15% (95% CI 0.26%-7.55%) in polypoid adenomas (p = 1, Figure 2, Table S2A and S3). These percentages are similar to the percentages found in the COSMIC database for these nucleotide positions (Table S1A and S2A). Of the five adenomas with a *BRAF* mutation, three were detected in a serrated phenotype (histologically, one was a traditional serrated adenoma (TSA) and two were sessile serrated lesion (SSL), all three were classified as a LST-F subtype). Four were found in adenomas proximally located, which included these three serrated flat adenomas (Table S3).

### NRAS, FBXW7 and CTNNB1

Two *NRAS* mutations were found, all in the distal colon, c.37G>C (p.G13R) in polypoid adenomas 1.08% (95% CI 0.03%-5.85%) and c.182A>G (p.Q61R) in flat adenomas 0.94% (95% CI 0.02%-5.14%). Both have not been described in adenomas before (Figure 2, Table S2B and S3). In the COSMIC database 2.8% *NRAS* mutations have been reported in adenomas (based on 142 samples, Figure 1, Table S1B).

Four *FBXW7* mutations were discovered, one in a flat adenoma (c.1394G>A (p.R465H)) and three in polypoid adenomas, namely c.832C>T (p.R278*), and two times c.1513C>T (p.R505C). This last mutation has not been described before in adenomas. One of the polypoid adenomas with a c.1513C>T (p.R505C) *FBXW7* mutation also harbors a *KRAS* mutation, (c.35G>A) (Table S2J and S3). The *FBXW7* mutation frequency was 3.23% (95% CI 0.67%-9.14%) for polypoid adenomas and 1.13% (95% CI 0.07%-5.44%) for flat adenomas (Figure 2, Table S2J) which was similar to reported frequencies in the COSMIC database (4.14% based on 145 samples, Figure 1, Table S1J and S2J ). One polypoid and one flat adenoma harbored a *CTNNB1* mutation, both at the same nucleotide position c.134C>T (p.S45F) and both adenomas contained no *APC* truncating mutation. One of the *CTNNB1* mutations was found in a sample that also showed a *KRAS* (c.35G>A) mutation (Table S3). The *CTNNB1* mutation frequency was 1.08% (95% CI 0.03%-5.85%) for polypoid adenomas and 0.94% (95% CI 0.02%-5.14%) for flat adenomas (Figure 2, Table S2K), giving no significant differences between the two phenotypes. The percentages found were in the same range as for the colorectal adenomas described in the COSMIC database (based on 964 samples, Figure 1, Table S1K and S2K).

### APC

According to the COSMIC database, the MCR harbors 76.37% of all the mutations described in the *APC* gene for adenomas (Figure 1, Table S1N). In the current study the position c.4348C>T (p.R1450*) was the most frequently mutated site. The vast majority of mutations found in this study are predicted to result in a premature stop codon (truncating mutation), except for four silent mutations and four missense mutations (Table S2N and S3). Two of these mutations were found in polypoid adenomas and six in flat adenomas. The truncating mutations involved 15 nucleotide substitutions, 5 nucleotide(s) insertions and 25 nucleotide(s) deletions for polypoid adenomas and 13 nucleotide substitutions, 3 nucleotide(s) insertions and 8 nucleotide(s) deletions for flat adenomas. Four samples contained a double mutation in *APC*, i.e. three flat adenomas and one polypoid adenoma. Three of these samples harbored a missense mutation (c.4326T>A (p.P1442P)) next to a truncating mutation and one flat adenoma contained two truncating mutations (c.4259_4272del14 (p.P1420fs*2) and c.4348C>T (p.R1450*) (Table S2N and S3)).

Overall, the mutation rate for truncating mutations in *APC* was significantly higher in polypoid adenomas compared to flat adenomas (48.49% (95% CI 37.23%-59.87%) versus 30.27% (95% CI 20.13%-42.05%), respectively, p = 0.02, odds ratio 0.46, 95% CI 0.25%-0.90%, Figure 2, Table S2N).

### Relationship between APC Mutation and Adenoma Location

After stratification for anatomical location the difference in *APC* mutation frequency between flat adenomas and polypoid adenomas persisted for the distal colon with 61.7% (37/60) of polypoid adenomas showing *APC* mutations compared to 31.0% (9/29) of flat adenomas. In the proximal colon, however, this difference could not be detected (33.3% (6/18) in polypoid adenomas versus 31.1% (14/45) in flat adenomas).

The exact mutation positions in the *APC* gene, result into different numbers of β-catenin down-regulating motifs (20 amino acid repeats) in the truncated protein. Mutations found in the proximal colon lead to APC proteins that in 15% (3/20) harbor one repeat and in 85% (17/20) two repeats whereas in the distal colon these figures were exactly fifty/fifty (23/46, harbored one repeat, and 23/46 harbored two repeats, proximal versus distal, p = 0.007). However, the number of repeats did not differ between flat and polypoid adenomas, also not after stratification for location.

### Relationship of APC Mutation and Subtypes of Flat Adenomas

Types IIa, LST-G and LST-F flat adenomas all contained significantly fewer *APC* mutations compared to polypoid adenomas (IIa: 29.6% (8/27), LST-G: 25% (3/12), LST-F: 34.4% (11/32) vs polypoid 55.6% (45/81) p = 0.02, 0.05 and 0.04, respectively). Of the three successfully annotated IIc type flat adenomas, one showed a mutation (c.4285C>T (p.Q1429*)) (Table S3).

Between the different subtypes, there was no difference in *APC* mutation frequency, although mutations in subtype IIa did not include deletions and mutations in both LST types did not include insertions (IIa contained 6 substitutions and 3 insertions, LST contained 12 substitutions and 8 deletions, p = 0.006). There was no difference found between the amount of β-catenin down-regulating repeats left in the truncated APC protein between the different subtypes.

### MSI

In 153 out of 199 adenomas (80 polypoid and 73 flat adenomas) MSI status could be successfully determined. Of these, only one flat adenoma (type IIa) showed MSI, no mutations or a specific phenotype were associated with this adenoma (Table S3).

## Discussion

Awareness of the clinical relevance of flat colorectal adenomas has grown over the last two decades [6]. As a consequence multiple studies have explored their pathogenesis. Some of these studies have suggested a different biology for flat adenomas, which was contradicted by other studies. Often, these claims were based on a limited sample size and many studies used suboptimal classification [29], [30]. Moreover, many studies had limited sample size, which hampers generalization of the findings [30], [31]. The aim of the present study was to investigate in parallel, in a large multi-centre well-defined series of flat and polypoid adenomas, the mutation status of 14 genes known to be involved in CRC. The genes studied are involved in a number of different signal transduction pathways.

### RAS-RAF-MAPK Pathway

The RAS-RAF-MAPK pathway is a key signal transduction pathway in many cancers including CRC and was investigated in the current study by examining the mutations status of *KRAS*, *NRAS*, *BRAF* and *MAP2K4*. In the context of flat colorectal lesions this pathway is still controversial, since it has been reported that *KRAS* mutations would be uncommon in flat adenomas [13], [14], while later studies claimed the opposite [16] or did not detect a difference with polypoid lesions [15], [32]. The present study involving a large well-defined sample series, supports these latter studies which do not detect a significant difference between the two phenotypes. Whilst the current study is the first to investigate *KRAS* mutations in codons 12, 13, 59 and 61 covering more than 97% of all reported mutations for *KRAS* in CRC, earlier studies investigated only codon 12 of the *KRAS* gene [13], [33]. Based on our results this could explain the reported differences for *KRAS* in flat adenomas observed in previous studies, since in the current study a significant difference for codon 12 was observed while for the whole *KRAS* gene this difference was absent. Other factors, however, such as sample selection and different flat adenoma subtypes could also influences the results observed in the previous studies. *KRAS* mutations have previously been reported to be less frequent in depressed (0-IIc) lesions [13], [29] compared to other flat subtypes and or polypoid lesions which is consistent with the current study, where non of the four IIc adenomas showed a *KRAS* mutation. Several studies suggested that *KRAS* mutations were significantly more frequent in LST-G than in LST-F cases [32], [34]. In the present study, the number of LST-G adenomas investigated was too small to draw a general conclusion. There was, however, a trend towards a higher mutation frequency in LST-G type adenomas compared to the LST-F types (34.67% versus 26.73%).

In literature the *NRAS* mutation rate in CRC is around 3% [35], [36]. One study has investigated *NRAS* mutation frequency in a very small series of flat adenomas showing a percentage of 25% (2 out of 8 samples) [37]. The percentages in the present study are around 1% in both flat and polypoid adenomas.

The current study confirms that *BRAF* and *KRAS* are mutually exclusive [38] and that *BRAF* mutations are associated with the serrated phenotype [39]. The *BRAF* mutation frequency of polypoid and flat adenomas was similar to the reported literature [16], [29], [32]. Whereas *BRAF* mutations are relatively more common in MSI colorectal carcinomas, for adenomas this is less straightforward [40]. As we reported only one MSI adenoma (similar to previous findings [14], [41]) we did not find an association between *BRAF* mutations and MSI.

Overall, when combining all the gene mutations of the RAS-RAF-MAPK pathway, no significant differences were detected between flat adenomas and polypoid colorectal adenomas.

### PI3K-AKT Pathway

In the current study, no mutations were found in the *PI3KCA* and *PIK3R1* nor in the negative regulator of the PI3K-AKT pathway, the *PTEN* gene. The mutation frequency in carcinomas for *PIK3CA* varies between 10–30% [42], [43] and mostly affects exons 9 and 20 (both completely covered in our analysis). In colorectal adenomas, however, *PIK3CA* mutations are less common, with mutation frequencies around 3%, indicating that mutations in *PIK3CA* generally would arise late in tumorigenesis [42]. Furthermore, these mutations have been reported to be uncommon (0 to 3%) in flat lesions and without significant difference compared to polypoid adenomas [29], [32]. Together these results indicate that mutation-driven activation of the PI3K-AKT pathway rarely occurs in polypoid adenomas as well as in flat adenomas.

### Cyclin Dependent Kinases

Cyclins, together with cyclin-dependent kinases, regulate the cell cycle. Disruption of cyclin activity can lead to cell cycle arrest or uncontrolled cell cycle progression. FBXW7 is a component of a ubiquitin ligase complex (F-box complex) which targets among others cyclin E and MYC, for degradation. Mutations in *FBXW7* are often missense mutations, mostly found in the WD40 domain [44]. Two of the mutations (R505C and R465C) described in the current study are in this domain and one (R275*) is outside this domain, in a non-sense mutation cluster (aa 220–393) [45].

### Wnt-signaling

Mutations in *CTNNB1* (β-catenin) have been reported as an alternative to *APC* mutations [12] and were found more frequently in small colorectal adenomas [46]. However, more recent studies fail to confirm this [47]. In the current study two mutations were reported, one in a small and one in a large adenoma. No significant difference between polypoid and flat adenomas was detected, which is in line with earlier findings [29].

In the present study, one of the largest investigating *APC* mutations in flat adenomas, a significantly lower frequency of *APC* truncating mutations was observed in flat adenomas compared to polypoid adenomas (30.27% versus 48.49%, respectively) similar to an earlier observations in a smaller sample set investigating depressed adenomas [13]. In the present series none of the mutations in subtype IIa were caused due to nucleotide deletions, which therefore would indicate these deletions to be rare in this type of lesions. However, Umetani *et al*, did find *APC* mutations due to nucleotide deletions, which contradicts this observation [13]. Between the different subtypes of flat lesions there was no significant difference and all showed lower mutation frequencies compared to polypoid adenomas.

In the current study two adenomas harbored a double mutation. Double mutations are not unique and are frequently reported in the literature especially in familial adenomatous polyposis (FAP) patients [48], [49].

Originally, all mutations in *APC* were considered to be equal in their impact on tumorigenesis. However, the “just-right” signaling model shows that there is a strong selective pressure on the level of β-catenin signaling resulting from specific *APC* mutations (i.e. depending on the mutation side, the truncated APC protein contains different amounts of β-catenin degradation repeats) [48]. In addition, it has been shown that the position of mutations in the *APC* gene differ along the GI tract [48] and that mutations at different positions yield different phenotypes in FAP patients [50]. In the current study a difference between left and right colon was observed, but no differences between flat and polypoid adenomas were observed.

From this study it appears that *APC* mutations and thus possibly the Wnt-signaling in general, would play a less prominent role in flat adenomas than in polypoid adenomas. However, alternative modes of Wnt activation, like mutations outside the MCR of *APC* (not investigated in the present study), promoter CpG-island methylation of *APC* or other Wnt related genes, or altered expression of relevant microRNAs cannot be excluded [51], [52]. Interestingly, we found that loss of chromosome 5q is significantly more frequent in flat adenomas compared to polypoid adenomas, which also may affect *APC* located within this loss. At the same time we observed fewer *APC* mutations in flat adenomas, compared to polypoid adenomas [11]. Thus the picture of mechanisms of APC disruption in flat adenomas may be more complex than in polypoid adenomas. As a consequence these alternative APC disrupting mechanisms that occur in flat lesions may also have different effects on molecular processes that are involved in the pathogenesis of CRC, such as stability of the cytoskeleton [53], or chromosomal instability [54]–[56], resulting in a more aggressive behavior of flat lesions.

Despite the relatively large amount of flat adenomas in the current study, the number of lesions from specific subtypes (i.e. IIc and LST-G) was relatively low, which may be due to the fact that overall these subtypes are less common. Therefore strong conclusions about potential differences in mutation frequencies in one of the subtypes cannot be drawn from this study.

All the mutations investigated in the current study have been described as somatic mutations (in the COMSMIC database), however the presence of germ line mutations cannot be excluded due to the lack of matched normal DNA.

In conclusion, besides *APC*, no significant differences were found for the investigated mutations between flat adenomas and polypoid adenomas. In contrast to previous observations, the RAS-RAF-MAPK pathway was equally affected by mutations in both flat adenomas and polypoid adenomas. Although, flat adenomas showed a lower frequency of *APC* mutations compared to polypoid adenomas, they were previously found to have higher frequency of chromosome 5q loss, including the *APC* gene locus. Therefore the difference in phenotype between flat and polypoid adenomas may be associated with alternative mechanisms of disrupting the Wnt-pathway.

## Supporting Information

Table S1 **Frequency of A)** ***BRAF*** **, B)** ***NRAS*** **, C)** ***KRAS*** **, D)** ***PIK3CA*** **, E)** ***PIK3R1*** **, F)** ***EGFR*** **, G)** ***PTEN*** **, H)** ***MAP2K4*** **, I)** ***SMAD4*** **, J)** ***FBXW7*** **, K)** ***CTNNB1*** **, L)** ***STK11*** **, M)** ***PDGFRA*** **and N)** ***APC*** **mutations according to the COSMIC database for carcinomas and adenomas.** -; no data available. Relative mutation distribution  =  percentage of specific mutation within the mutation subpopulation. Absolute mutation frequency  =  percentage of specific mutations in the whole study population  =  incidence. Grey blocks represent excluded assays.
(DOC)

Table S2 **Frequency of A)** ***BRAF*** **, B)** ***NRAS*** **, C)** ***KRAS*** **, D)** ***PIK3CA*** **, E)** ***PIK3R1*** **, F)** ***EGFR*** **, G)** ***PTEN*** **, H)** ***MAP2K4*** **, I)** ***SMAD4*** **, J)** ***FBXW7*** **, K)** ***CTNNB1*** **, L)** ***STK11*** **, M)** ***PDGFRA*** **and N)** ***APC*** **mutations in the flat and polypoid adenomas.** CI; 95% confidence interval, ^#^; number of samples where all assays succeeded, ^##^; mutation percentage based on the random input algorithm, ^###^; p-value calculated based on random impute algorithm.
(DOC)

Table S3 **Overview of all mutated samples.** MSS; microsatelite stable, MSI; microsatelite instable, SSL; sessile serrated lesion, TSA; traditional serrated adenoma, *Italic*; silent mutation, ^#^;Mutation not described before in colorectal adenomas, -; no data available.
(DOC)
