# Supplementary material for: Comprehensive Mutation Analysis in Colorectal Flat Adenomas
Source: PLoS One. 2012 Jul 27;7(7):e41963. doi: 10.1371/journal.pone.0041963 (PMC3407043; doi:10.1371/journal.pone.0041963)
Supplement: Table S1 — Frequency of A) BRAF , B) NRAS , C) KRAS , D) PIK3CA , E) PIK3R1 , F) EGFR , G) PTEN , H) MAP2K4 , I) SMAD4 , J) FBXW7 , K) CTNNB1 , L) STK11 , M) PDGFRA and N) APC mutations according to the COSMIC database for carcinomas and adenomas. -; no data available. Relative mutation distribution = percentage of specific mutation within the mutation subpopulation. Absolute mutation frequency = percentage of specific mutations in the whole study population = incidence. Grey blocks represent excluded assays. (DOC) [file pone.0041963.s001.doc]

| **GENE** | **AMINO ACID CHANGE** | **NUCLEOTIDE MUTATION** | **COSMIC RELATIVE MUTATION DITRIBUTION CARCINOMAS % (N)** | **COSMIC ABSOLUTE MUTATION FREQUENCY CARCINOMAS %** |  | **COSMIC RELATIVE MUTATION DITRIBUTION ADENOMAS % (N)** | **COSMIC ABSOLUTE MUTATION FREQUENCY ADENOMAS %** |
| --- | --- | --- | --- | --- | --- | --- | --- |
| ***BRAF*** | p.D594G | c.1781A>G | 0.37 (11) | 0.04 |  | 0 (0) | 0 |
|  | p.V600E | c.1799T>A | 97.38 (2862) | 10.05 |  | 88.64 (39) | 2.85 |
|  | p.V600M | c.1798G>A | 0.03 (1) | 0 |  | 0 (0) | 0 |
|  | p.K601E | c.1801A>G | 0.14 (4) | 0.01 |  | 2.27 (1) | 0.07 |
|  | **TOTAL MUTANTS (SEQUENOM)** | | 97.92 (2878) | 10.1 |  | 90.91 (40) | 2.92 |
|  | **TOTAL MUTANTS (COSMIC)** | | 100 (2940) | 10.32 |  | 100 (44) | 3.21 |
|  |  |  |  |  |  |  |  |
| **GENE** | **AMINO ACID CHANGE** | **NUCLEOTIDE MUTATION** | **COSMIC RELATIVE MUTATION DITRIBUTION CARCINOMAS % (N)** | **COSMIC ABSOLUTE MUTATION FREQUENCY CARCINOMAS %** |  | **COSMIC RELATIVE MUTATION DITRIBUTION ADENOMAS % (N)** | **COSMIC ABSOLUTE MUTATION FREQUENCY ADENOMAS %** |
| ***NRAS*** | p.G12C | c.34G>T | 12.5 (2) | 0.31 |  | 25 (1) | 0.7 |
|  | p.G12S | c.34G>A | 6.25 (1) | 0.16 |  | 0 (0) | 0 |
|  | p.G12R | c.34G>C | 0 (0) | 0 |  | 0 (0) | 0 |
|  | p.G13R | c.37G>C | 6.25 (1) | 0.16 |  | 0 (0) | 0 |
|  | p.G13S | c.37G>A | 0 (0) | 0 |  | 0 (0) | 0 |
|  | p.G13C | c.37G>T | 0 (0) | 0 |  | 0 (0) | 0 |
|  | p.Q61K | c.181C>A | 0 (0) | 0 |  | 75 (3) | 2.11 |
|  | p.Q61E | c.181C>G | 0 (0) | 0 |  | 0 (0) | 0 |
|  | p.Q61R | c.182A>G | 37.5 (6) | 0.93 |  | 0 (0) | 0 |
|  | p.Q61P | c.182A>C | 0 (0) | 0 |  | 0 (0) | 0 |
|  | p.Q61L | c.182A>T | 6.25 (1) | 0.16 |  | 0 (0) | 0 |
|  | p.Q61H | c.183A>C | 0 (0) | 0 |  | 0 (0) | 0 |
|  | p.Q61Q | c.183A>G | 0 (0) | 0 |  | 0 (0) | 0 |
|  | p.Q61H | c.183A>T | 0 (0) | 0 |  | 0 (0) | 0 |
|  | TOTAL CODON 12 |  | 18.75 (3) | 0.47 |  | 25 (1) | 0.7 |
|  | TOTAL CODON 13 |  | 6.25 (1) | 0.16 |  | 0 (0) | 0 |
|  | TOTAL CODON 61 |  | 43.75 (7) | 1.09 |  | 75 (3) | 2.11 |
|  | **TOTAL MUTANTS (SEQUENOM)** | | 68.75 (11) | 1.71 |  | 100 (4) | 2.82 |
|  | **TOTAL MUTANTS (COSMIC)** | | 100 (16) | 2.48 |  | 100 (4) | 2.82 |

Supplementary table S1A and S1B

| **GENE** | **AMINO ACID CHANGE** | **NUCLEOTIDE MUTATION** | **COSMIC RELATIVE MUTATION DITRIBUTION CARCINOMAS % (N)** | **COSMIC ABSOLUTE MUTATION FREQUENCY CARCINOMAS %** |  | **COSMIC RELATIVE MUTATION DITRIBUTION ADENOMAS % (N)** | **COSMIC ABSOLUTE MUTATION FREQUENCY ADENOMAS %** |
| --- | --- | --- | --- | --- | --- | --- | --- |
| ***KRAS*** | p.G12C | c.34G>T | 7.99 (600) | 2.84 |  | 9.96 (69) | 2.19 |
|  | p.G12S | c.34G>A | 5.97 (448) | 2.12 |  | 6.2 (43) | 1.37 |
|  | p.G12R | c.34G>C | 1.17 (88) | 0.42 |  | 2.6 (18) | 0.57 |
|  | p.G12D | c.35G>A | 33.97 (2551) | 12.06 |  | 31.6 (219) | 6.95 |
|  | p.G12V | c.35G>T | 21.15 (1588) | 7.51 |  | 32.47 (225) | 7.14 |
|  | p.G12A | c.35G>C | 6.56 (493) | 2.33 |  | 4.76 (33) | 1.05 |
|  | p.G13D | c.38G>A | 19.23 (1444) | 6.83 |  | 10.25 (71) | 2.25 |
|  | p.G13A | c.38G>C | 0.09 (7) | 0.03 |  | 0.29 (2) | 0.06 |
|  | p.G13V | c.38G>T | 0.12 (9) | 0.04 |  | 0.14 (1) | 0.03 |
|  | p.G13G | c.39C>A | 0.03 (2) | 0.01 |  | 0 (0) | 0 |
|  | p.G13G | c.39C>G | 0.01 (1) | 0 |  | 0 (0) | 0 |
|  | p.G13G | c.39C>T | 0 (0) | 0 |  | 0 (0) | 0 |
|  | p.A59T | c.175G>A | 0.01 (1) | 0 |  | 0 (0) | 0 |
|  | p.Q61K | c.181C>A | 0.05 (4) | 0.05 |  | 0 (0) | 0 |
|  | p.Q61E | c.181C>G | 0.01 (1) | 0.01 |  | 0 (0) | 0 |
|  | p.Q61L | c.182A>T | 0.15 (11) | 0.15 |  | 0 (0) | 0 |
|  | p.Q61R | c.182A>G | 0.08 (6) | 0.08 |  | 0 (0) | 0 |
|  | p.Q61P | c.182A>C | 0 (0) | 0 |  | 0 (0) | 0 |
|  | p.Q61H | c.183A>C | 0.32 (24) | 0.32 |  | 0.43 (3) | 0.1 |
|  | p.Q61H | c.183A>T | 0.2 (15) | 0.2 |  | 0.14 (1) | 0.03 |
|  | p.A146T | c.436G>A | 0.27 (20) | 0.09 |  | 0 (0) | 0 |
|  | p.A146P | c.436G>C | 0.03 (2) | 0.01 |  | 0 (0) | 0 |
|  | TOTAL CODON 12 |  | 76.81 (5768) | 27.28 |  | 87.59 (607) | 19.27 |
|  | TOTAL CODON 13 |  | 19.48 (1463) | 6.91 |  | 10.68 (74) | 2.34 |
|  | TOTAL CODON 61 |  | 0.81 (61) | 0.81 |  | 0.57 (4) | 0.13 |
|  | TOTAL CODON 146 |  | 0.30 (22) | 0.1 |  | 0 (0) | 0 |
|  | **TOTAL MUTANTS (SEQUENOM)** | | 97.28 (7306) | 34.54 |  | 98.85 (685) | 21.75 |
|  | **TOTAL MUTANTS (COSMIC)** | | 100 (7511) | 35.51 |  | 100 (693) | 22 |

Supplementary table S1C

| **GENE** | **AMINO ACID CHANGE** | **NUCLEOTIDE MUTATION** | **COSMIC RELATIVE MUTATION DITRIBUTION CARCINOMAS % (N)** | **COSMIC ABSOLUTE MUTATION FREQUENCY CARCINOMAS %** | **EXON** | **COSMIC RELATIVE MUTATION DITRIBUTION ADENOMAS % (N)** | **COSMIC ABSOLUTE MUTATION FREQUENCY ADENOMAS %** |
| --- | --- | --- | --- | --- | --- | --- | --- |
| ***PIK3CA*** | p.G12D | c.35G>A | 0.16 (1) | 0.02 | 1 | 0 (0) | 0 |
|  | p.R38H | c.113G>A | 0.47 (3) | 0.07 | 1 | 0 (0) | 0 |
|  | p.E81K | c.241G>A | 0.16 (1) | 0.02 | 1 | 0 (0) | 0 |
|  | p.R88Q | c.263G>A | 0.95 (6) | 0.13 | 1 | 0 (0) | 0 |
|  | p.R93W | c.277C>T | 0.47 (3) | 0.07 | 1 | 0 (0) | 0 |
|  | p.G106V | c.317G>T | 0.47 (3) | 0.07 | 1 | 0 (0) | 0 |
|  | p.R108H | c.323G>A | 0.63 (4) | 0.63 | 1 | 0 (0) | 0 |
|  | p.G118D | c.353G>A | 0.47 (3) | 0.47 | 2 | 0 (0) | 0 |
|  | p.P134S | c.400C>T | 0.16 (1) | 0.02 | 2 | 0 (0) | 0 |
|  | p.S158L | c.473C>T | 0.16 (1) | 0.02 | 2 | 0 (0) | 0 |
|  | p.H160N | c.478C>A | 0.16 (1) | 0.02 | 2 | 0 (0) | 0 |
|  | p.K179T | c.536A>C | 0.16 (1) | 0.02 | 2 | 0 (0) | 0 |
|  | p.K184E | c.550A>G | 0.16 (1) | 0.02 | 2 | 0 (0) | 0 |
|  | p.N345K | c.1035T>A | 0.47 (3) | 0.07 | 4 | 0 (0) | 0 |
|  | p.C420R | c.1258T>C | 0.79 (5) | 0.11 | 7 | 0 (0) | 0 |
|  | p.P539R | c.1616C>G | 0.16 (1) | 0.02 | 9 | 0 (0) | 0 |
|  | p.E542K | c.1624G>A | 15.8 (100) | 2.17 | 9 | 25 (1) | 0.44 |
|  | p.E542Q | c.1624G>C | 0.32 (2) | 0.04 | 9 | 0 (0) | 0 |
|  | p.E545K | c.1633G>A | 28.28 (179) | 3.88 | 9 | 25 (1) | 0.44 |
|  | p.Q546K | c.1636C>A | 3.95 (25) | 0.54 | 9 | 0 (0) | 0 |
|  | p.Q546E | c.1636C>G | 0.47 (3) | 0.07 | 9 | 0 (0) | 0 |
|  | p.H701P | c.2102A>C | 0 (0) | 0 | 13 | 0 (0) | 0 |
|  | p.C901F | c.2702G>T | 0.16 (1) | 0.02 | 18 | 0 (0) | 0 |
|  | p.M1004I | c.3012G>T | 0.16 (1) | 0.02 | 20 | 0 (0) | 0 |
|  | p.G1007R | G3019C | 0.16 (1) | 0.02 | 20 | 0 (0) | 0 |
|  | p.H1047Y | c.3139C>T | 1.42 (9) | 0.2 | 20 | 0 (0) | 0 |
|  | p.H1047R | c.3140A>G | 19.12 (121) | 2.62 | 20 | 0 (0) | 0 |
|  | p.H1047L | c.3140A>T | 3.48 (22) | 0.48 | 20 | 0 (0) | 0 |
|  | p.G1049S | c.3145G>A | 0 (0) | 0 | 20 | 25 (1) | 0.44 |
|  | p.G1049R | c.3145G>C | 2.53 (16) | 0.35 | 20 | 0 (0) | 0 |
|  | exon 9 |  | 49.43 (310) | 6.72 |  | 50 (2) | 0.88 |
|  | exon 20 |  | 26.87 (170) | 3.69 |  | 25 (1) | 0.44 |
|  | **TOTAL MUTANTS (SEQUENOM)** | | 81.36 (515) | 11.17 |  | 75 (3) | 1.32 |
|  | **TOTAL MUTANTS (COSMIC)** | | 100 (633) | 13.73 |  | 100 (4) | 1.78 |

Supplementary table S1D

| **GENE** | **AMINO ACID CHANGE** | **NUCLEOTIDE MUTATION** | **COSMIC RELATIVE MUTATION DITRIBUTION CARCINOMAS % (N)** | **COSMIC ABSOLUTE MUTATION FREQUENCY CARCINOMAS %** |  | **COSMIC RELATIVE MUTATION DITRIBUTION ADENOMAS % (N)** | **COSMIC ABSOLUTE MUTATION FREQUENCY ADENOMAS %** |
| --- | --- | --- | --- | --- | --- | --- | --- |
| ***PIK3R1*** | p.N564K | C/G | 0 (0) | 0 |  | - | - |
|  | p.W583del | GGT/del | 0 (0) | 0 |  | - | - |
|  | **TOTAL MUTANTS (SEQUENOM)** | | 0 (0) | 0 |  | - | - |
|  | **TOTAL MUTANTS (COSMIC)** | | 100 (3) | 3.85 |  |  |  |
|  |  |  |  |  |  |  |  |
| **GENE** | **AMINO ACID CHANGE** | **NUCLEOTIDE MUTATION** | **COSMIC RELATIVE MUTATION DITRIBUTION CARCINOMAS % (N)** | **COSMIC ABSOLUTE MUTATION FREQUENCY CARCINOMAS %** |  | **COSMIC RELATIVE MUTATION DITRIBUTION ADENOMAS % (N)** | **COSMIC ABSOLUTE MUTATION FREQUENCY ADENOMAS %** |
| ***EGFR*** | p.E746_A750del | c.2235_2249del15 | 0 (0) | 0 |  | - | - |
|  | p.E746_A750del | c.2236_2250del15 | 0 (0) | 0 |  | - | - |
|  | **TOTAL MUTANTS (SEQUENOM)** | | 0 (0) | 0 |  | - | - |
|  | **TOTAL MUTANTS (COSMIC)** | | 100 (8) | 0.68 |  |  |  |
|  |  |  |  |  |  |  |  |
| **GENE** | **AMINO ACID CHANGE** | **NUCLEOTIDE MUTATION** | **COSMIC RELATIVE MUTATION DITRIBUTION CARCINOMAS % (N)** | **COSMIC ABSOLUTE MUTATION FREQUENCY CARCINOMAS %** |  | **COSMIC RELATIVE MUTATION DITRIBUTION ADENOMAS % (N)** | **COSMIC ABSOLUTE MUTATION FREQUENCY ADENOMAS %** |
| ***PTEN*** | p.E150Q | c.448G>C | 1.69 (1) | 0.22 |  | 0 (0) | 0 |
|  | p.F241S | c.722T>C | 1.69 (1) | 0.22 |  | 0 (0) | 0 |
|  | p.K267fs*9 | c.800delA | 22.03 (13) | 2.91 |  | 0 (0) | 0 |
|  | p.K62R | c.185A>G | 1.69 (1) | 0.22 |  | 0 (0) | 0 |
|  | p.Y65C | c.194A>G | 1.69 (1) | 0.22 |  | 0 (0) | 0 |
|  | **TOTAL MUTANTS (SEQUENOM)** | | 28.81 (17) | 3.81 |  | 0 (0) | 0 |
|  | **TOTAL MUTANTS (COSMIC)** | | 100 (59) | 13.23 |  | 100 (1) | 0 |
|  |  |  |  |  |  |  |  |
| **GENE** | **AMINO ACID CHANGE** | **NUCLEOTIDE MUTATION** | **COSMIC RELATIVE MUTATION DITRIBUTION CARCINOMAS % (N)** | **COSMIC ABSOLUTE MUTATION FREQUENCY CARCINOMAS %** |  | **COSMIC RELATIVE MUTATION DITRIBUTION ADENOMAS % (N)** | **COSMIC ABSOLUTE MUTATION FREQUENCY ADENOMAS %** |
| ***MAP2K4*** | p.Q142L | c.425A>T | 0 (0) | 0 |  | - | - |
|  | p.R154W | c.460C>T | 22.22 (2) | 1.12 |  | - | - |
|  | p.E221* | c.661G>T | 0 (0) | 0 |  | - | - |
|  | p.S251N | c.752G>A | 0 (0) | 0 |  | - | - |
|  | p.S280* | c.839C>A | 0 (0) | 0 |  | - | - |
|  | p.I295fs*23 | c.882_882delG | 0 (0) | 0 |  | - | - |
|  | p.K309N | c.927G>C | 0 (0) | 0 |  | - | - |
|  | **TOTAL MUTANTS (SEQUENOM)** | | 22.22 (2) | 1.12 |  |  |  |
|  | **TOTAL MUTANTS (COSMIC)** | | 100 (9) | 5.06 |  |  |  |

Supplementary table S1E, S1F, S1G and S1H

|  |  |  |  |  |  |  |  |
| --- | --- | --- | --- | --- | --- | --- | --- |
| **GENE** | **AMINO ACID CHANGE** | **NUCLEOTIDE MUTATION** | **COSMIC RELATIVE MUTATION DITRIBUTION CARCINOMAS % (N)** | **COSMIC ABSOLUTE MUTATION FREQUENCY CARCINOMAS %** |  | **COSMIC RELATIVE MUTATION DITRIBUTION ADENOMAS % (N)** | **COSMIC ABSOLUTE MUTATION FREQUENCY ADENOMAS %** |
| ***SMAD4*** | p.E330A | c.989A>C | 2.04 (2) | 1.2 |  | 0 (0) | 0 |
|  | p.D351N | c.1051G>A | 1.02 (1) | 0.6 |  | 0 (0) | 0 |
|  | p.D351H | c.1051G>C | 3.06 (3) | 1.81 |  | 0 (0) | 0 |
|  | p.G386R | c.1156G>C | 5.10 (5) | 3.01 |  | 0 (0) | 0 |
|  | p.A433V | c.1298C>T | 1.02 (1) | 0.6 |  | 0 (0) | 0 |
|  | p.R445* | c.1333C>T | 2.04 (2) | 1.2 |  | 0 (0) | 0 |
|  | p.Q245* | c.733C>T | 0 (0) | 0 |  | 0 (0) | 0 |
|  | **TOTAL MUTANTS (SEQUENOM)** | | 14.29 (14) | 8.43 |  | 0 (0) | 0 |
|  | **TOTAL MUTANTS (COSMIC)** | | 100 (44) | 14.29 |  | 100 (3) | 13.64 |
|  |  |  |  |  |  |  |  |

Supplementary table S1I

| **GENE** | **AMINO ACID CHANGE** | **NUCLEOTIDE MUTATION** | **COSMIC RELATIVE MUTATION DITRIBUTION CARCINOMAS % (N)** | **COSMIC ABSOLUTE MUTATION FREQUENCY CARCINOMAS %** |  | **COSMIC RELATIVE MUTATION DITRIBUTION ADENOMAS % (N)** | **COSMIC ABSOLUTE MUTATION FREQUENCY ADENOMAS %** |
| --- | --- | --- | --- | --- | --- | --- | --- |
| ***FBXW7*** | p.R278* | c.832C>T | 6.76 (5) | 0.62 |  | 16.67 (1) | 0.69 |
|  | p.R393* | c.1177C>T | 5.41 (4) | 0.5 |  | 0 (0) | 0 |
|  | p.R465C | c.1393C>T | 12.16 (9) | 1.11 |  | 16.67 (1) | 0.69 |
|  | p.R465H | c.1394G>A | 6.76 (5) | 0.62 |  | 16.67 (1) | 0.69 |
|  | p.R479Q | c.1436G>A | 9.46 (7) | 0.87 |  | 16.67 (1) | 0.69 |
|  | p.V504I | c.1510G>A | 1.35 (1) | 0.12 |  | 0 (0) | 0 |
|  | p.R505C | c.1513C>T | 9.46 (7) | 0.87 |  | 0 (0) | 0 |
|  | p.R505H | c.1514G>A | 1.35 (1) | 0.12 |  | 16.67 (1) | 0.69 |
|  | **TOTAL MUTANTS (SEQUENOM)** | | 52.71 (39) | 4.83 |  | 83.35 (5) | 3.45 |
|  | **TOTAL MUTANTS (COSMIC)** | | 100 (74) | 9.16 |  | 100 (6) | 4.14 |

Supplementary table S1J

|  |  |  |  |  |  |  |  |
| --- | --- | --- | --- | --- | --- | --- | --- |
| **GENE** | **AMINO ACID CHANGE** | **NUCLEOTIDE MUTATION** | **COSMIC RELATIVE MUTATION DITRIBUTION CARCINOMAS % (N)** | **COSMIC ABSOLUTE MUTATION FREQUENCY CARCINOMAS %** |  | **COSMIC RELATIVE MUTATION DITRIBUTION ADENOMAS % (N)** | **COSMIC ABSOLUTE MUTATION FREQUENCY ADENOMAS %** |
| ***CTNNB1*** | p.G34R | c.100G>A | 0.62 (1) | 0.04 |  | 2.5 (1) | 0.1 |
|  | p.S45F | c.134C>T | 24.84 (40) | 1.45 |  | 32.5 (13) | 1.35 |
|  | **TOTAL MUTANTS (SEQUENOM)** | | 25.46 (41) | 1.49 |  | 35 (14) | 1.36 |
|  | **TOTAL MUTANTS (COSMIC)** | | 100 (161) | 5.82 |  | 100 (40) | 4.15 |
|  |  |  |  |  |  |  |  |
| **GENE** | **AMINO ACID CHANGE** | **NUCLEOTIDE MUTATION** | **COSMIC RELATIVE MUTATION DITRIBUTION CARCINOMAS % (N)** | **COSMIC ABSOLUTE MUTATION FREQUENCY CARCINOMAS %** |  | **COSMIC RELATIVE MUTATION DITRIBUTION ADENOMAS % (N)** | **COSMIC ABSOLUTE MUTATION FREQUENCY ADENOMAS %** |
| ***STK11*** | p.Q170* | c.508C>T | 0 (0) | 0 |  | 0 (0) | 0 |
|  | **TOTAL MUTANTS (SEQUENOM)** | | 0 (0) | 0 |  | 0 (0) | 0 |
|  | **TOTAL MUTANTS (COSMIC)** | | 100 (10) | 2.51 |  | 100 (2) | 7.69 |

Supplementary table S1K and S1L

|  |  |  |  |  |  |  |  |
| --- | --- | --- | --- | --- | --- | --- | --- |
| **GENE** | **AMINO ACID CHANGE** | **NUCLEOTIDE MUTATION** | **COSMIC RELATIVE MUTATION DITRIBUTION CARCINOMAS % (N)** | **COSMIC ABSOLUTE MUTATION FREQUENCY CARCINOMAS %** |  | **COSMIC RELATIVE MUTATION DITRIBUTION ADENOMAS % (N)** | **COSMIC ABSOLUTE MUTATION FREQUENCY ADENOMAS %** |
| ***PDGFRA*** | p.D1071N | c.3211G>A | 0 (0) | 0 |  | - | - |
|  | p.D842V | c.2525A>T | 0 (0) | 0 |  | - | - |
|  | p.T674I | c.2021C>T | 0 (0) | 0 |  | - | - |
|  | p.V561D | c.1682T>A | 0 (0) | 0 |  | - | - |
|  | **TOTAL MUTANTS (SEQUENOM)** | | 0 (0) | 0 |  | - | - |
|  | **TOTAL MUTANTS (COSMIC)** | | 100 (0) | 0 |  | - | - |

Supplementary table S1M

| **GENE** | **AMINO ACID CHANGE** | **NUCLEOTIDE MUTATION** | **COSMIC RELATIVE MUTATION DITRIBUTION CARCINOMAS % (N)** | **COSMIC ABSOLUTE MUTATION FREQUENCY CARCINOMAS %** | **nr of 20 AA repeats** | **COSMIC RELATIVE MUTATION DITRIBUTION ADENOMAS % (N)** | **COSMIC ABSOLUTE MUTATION FREQUENCY ADENOMAS %** |
| --- | --- | --- | --- | --- | --- | --- | --- |
| ***APC*** | p.Q1291* | c.3871C>T | 0.68 (7) | 0.25 | 1 | 0 (0) | 0 |
|  | p.T1301fs*15 | c.3900_3901insT | 0 (0) | 0 | 1 | 0 (0) | 0 |
|  | p.L1302fs*3 | c.3903delC | 0.10 (1) | 0.04 | 1 | 0 (0) | 0 |
|  | p.I1304fs*4 | c.3912delA | 0.10 (1) | 0.04 | 1 | 0 (0) | 0 |
|  | p.E1306* | c.3916G>T | 0.78 (8) | 0.29 | 1 | 0.52 (4) | 0.28 |
|  | p.E1309fs*4 | c.3921_3925delAAAAG | 4.30 (44) | 1.59 | 1 | 2.22 (17) | 2.04 |
|  | p.E1309* | c.3925G>T | 1.17 (12) | 0.43 | 1 | 0.26 (2) | 0.14 |
|  | p.K1310* | c.3928A>T | 0.20 (2) | 0.07 | 1 | 0.13 (1) | 0.07 |
|  | p.S1315* | c.3944C>A | 0.39 (4) | 0.14 | 1 | 0.13 (1) | 0.07 |
|  | *p.E1317Q* | *c.3949G>C* | 0.20 (2) | 0.07 | 7 (all) | 0 (0) | 0 |
|  | p.V1320fs*11 | c.3957_3958insT | 0 (0) | 0 | 1 | 0 (0) | 0 |
|  | p.E1322* | c.3964G>T | 0.68 (7) | 0.25 | 1 | 0.91 (7) | 0.49 |
|  | p.R1331* | c.3991A>T | 0.20 (2) | 0.07 | 1 | 0 (0) | 0 |
|  | p.Q1338* | c.4012C>T | 1.17 (12) | 0.43 | 1 | 1.04 (8) | 0.56 |
|  | p.S1344* | c.4031C>A | 0 (0) | 0 | 1 | 0.13 (1) | 0.07 |
|  | p.E1353* | c.4057G>T | 0.59 (6) | 0.22 | 1 | 0 (0) | 0 |
|  | p.S1356* | c.4067C>G | 0.29 (3) | 0.11 | 1 | 0.26 (2) | 0.14 |
|  | p.Q1367* | c.4099C>T | 1.37 (14) | 0.51 | 1 | 0.26 (2) | 0.14 |
|  | p.Q1378fs*7 | c.4131_4132insT | 0 (0) | 0 | 1 | 0 (0) | 0 |
|  | p.Q1378* | c.4132C>T | 1.07 (11) | 0.4 | 1 | 3.66 (28) | 1.97 |
|  | p.E1397fs*1 | c.4184_4185insT | 0 (0) | 0 | 2 | 0.13 (1) | 0.07 |
|  | p.S1400* | c.4199C>A | 0.29 (3) | 0.11 | 2 | 0 (0) | 0 |
|  | p.Q1406fs*11 | c.4216_4217insCGTTC | 0 (0) | 0 | 2 | 0 (0) | 0 |
|  | p.Q1406* | c.4216C>T | 0.59 (6) | 0.22 | 2 | 0.26 (2) | 0.14 |
|  | p.E1408* | c.4222G>T | 0.49 (5) | 0.18 | 2 | 0.26 (2) | 0.14 |
|  | p.S1411fs*1 | c.4232_4238delGTG  GAAT | 0 (0) | 0 | 2 | 0 (0) | 0 |
|  | p.S1411fs*4 | c.4233delT | 0.59 (6) | 0.22 | 2 | 0.65 (5) | 0.35 |
|  | p.V1414fs*5 | c.4241delT | 0 (0) | 0 | 2 | 0.13 (1) | 0.07 |
|  | p.G1416fs*3 | c.4247delG | 0.10 (1) | 0.04 | 2 | 0 (0) | 0 |
|  | p.P1420fs*2 | c.4259_4272delCCC  AGTGATCTTCC | 0 (0) | 0 | 2 | 0 (0) | 0 |
|  | p.S1421fs*52 | c.4263delT | 0.20 (2) | 0.07 | 2 | 0 (0) | 0 |
|  | p.Q1429* | c.4285C>T | 0.78 (8) | 0.29 | 2 | 2.87 (22) | 1.55 |
|  | p.T1430fs*43 | c.4287delC | 0 (0) | 0 | 2 | 0 (0) | 0 |
|  | p.P1432fs*35 | c.4294_4314delCACC  AAGCAGAAGTAAAAC | 0 (0) | 0 | 2 | 0 (0) | 0 |
|  | p.T1438fs*35 | c.4312delA | 0.39 (4) | 0.14 | 2 | 0.52 (4) | 0.28 |
|  | p.T1438fs*35 | c.4313delC | 0 (0) | 0 | 2 | 0.13 (1) | 0.07 |
|  | p.P1439fs*34 | c.4316delC | 0.10 (1) | 0.04 | 2 | 0.26 (2) | 0.14 |
|  | p.P1441fs*32 | c.4322delC | 0 (0) | 0 | 2 | 0 (0) | 0 |
|  | *p.P1442P* | *c.4326T>A* | 0.20 (2) | 0.07 | 7 (all) | 0 (0) | 0 |
|  | p.T1445fs*28 | c.4333delA | 0 (0) | 0 | 2 | 0 (0) | 0 |
|  | p.T1445fs*28 | c.4334delC | 0 (0) | 0 | 2 | 0.13 (1) | 0.07 |
|  | p.R1450* | c.4348C>T | 5.86 (60) | 2.17 | 2 | 2.87 (22) | 1.55 |
|  | p.E1461* | c.4381G>T | 0.10 (1) | 0.04 | 2 | 0 (0) | 0 |
|  | p.E1461fs*7 | c.4382_4383insA | 0 (0) | 0 | 2 | 0 (0) | 0 |
|  | p.S1465fs*3 | c.4386_4387delGA | 1.86 (19) | 0.69 | 2 | 0.65 (5) | 0.35 |
|  | p.K1462fs*10 | c.4386delG | 0 (0) | 0 | 2 | 0 (0) | 0 |
|  | *p.P1483S* | *c.4447C>T* | 0 (0) | 0 | 7 (all) | 0 (0) | 0 |
|  | *p.A1485T* | *c.4453G>A* | 0 (0) | 0 | 7 (all) | 0 (0) | 0 |
|  | p.T1487fs*27 | c.4460_4464delCTTTA | 0 (0) | 0 | 2 | 0 (0) | 0 |
|  | p.L1488fs*19 | c.4464delA | 0.39 (4) | 0.14 | 2 | 0 (0) | 0 |
|  | p.H1490fs*17 | c.4469_4470insTA | 0 (0) | 0 | 2 | 0 (0) | 0 |
|  | p.A1492fs*22 | c.4473_4474insT | 0.20 (2) | 0.07 | 2 | 0 (0) | 0 |
|  | p.E1494fs*13 | c.4480delG | 0.29 (3) | 0.11 | 2 | 0.13 (1) | 0.07 |
|  | *p.G1499R* | *c.4495G>A* | 0 (0) | 0 | 7 (all) | 0 (0) | 0 |
|  | other mutations within MCR (1286-1513) | | 46.59 (477) | 17.24 |  | 56.27 (431) | 30.35 |
|  | **TOTAL MUTANTS (MCR)** | | 72.27 (740) | 26.75 |  | 76.37 (585) | 41.2 |
|  | **TOTAL MUTANTS (COSMIC)** | | 100 (1024) | 37.01 |  | 100 (766) | 53.94 |
|  |  |  |  |  |  |  |  |

Supplementary table S1N
