# Supplementary material for: Comprehensive Mutation Analysis in Colorectal Flat Adenomas
Source: PLoS One. 2012 Jul 27;7(7):e41963. doi: 10.1371/journal.pone.0041963 (PMC3407043; doi:10.1371/journal.pone.0041963)
Supplement: Table S2 — Frequency of A) BRAF , B) NRAS , C) KRAS , D) PIK3CA , E) PIK3R1 , F) EGFR , G) PTEN , H) MAP2K4 , I) SMAD4 , J) FBXW7 , K) CTNNB1 , L) STK11 , M) PDGFRA and N) APC mutations in the flat and polypoid adenomas. CI; 95% confidence interval, #; number of samples where all assays succeeded, ##; mutation percentage based on the random input algorithm, ###; p-value calculated based on random impute algorithm. (DOC) [file pone.0041963.s002.doc]

| **GENE** | **AMINO ACID CHANGE** | **NUCLEOTIDE MUTATION** | **FLAT ADENOMAS** | **POLYPOID ADENOMAS** | **P-value^###^** |
| --- | --- | --- | --- | --- | --- |
| ***BRAF*** | p.D594G | c.1781A>G | 0/102 | 0/93 |  |
|  | p.V600E | c.1799T>A | 3/105 | 2/93 |  |
|  | **completely succeeded ^#^** | | 3/101 | 2/93 |  |
|  | **percentage mutated ^##^** | | 2.83 (CI 0.59-8.05) | 2.15 (CI 0.26-7.55) | 1, odds ration 1.32, CI 0.20-10.87 |
|  |  |  |  |  |  |
| **GENE** | **AMINO ACID CHANGE** | **NUCLEOTIDE MUTATION** | **FLAT ADENOMAS** | **POLYPOID ADENOMAS** | **P-value^###^** |
| ***NRAS*** | p.G12C | c.34G>T | 0/78 | 0/82 |  |
|  | p.G12S | c.34G>A | 0/78 | 0/82 |  |
|  | p.G12R | c.34G>C | 0/78 | 0/82 |  |
|  | p.G13R | c.37G>C | 0/89 | 1/92 |  |
|  | p.G13S | c.37G>A | 0/89 | 0/92 |  |
|  | p.G13C | c.37G>T | 0/89 | 0/92 |  |
|  | p.Q61K | c.181C>A | 0/48 | 0/85 |  |
|  | p.Q61E | c.181C>G | 0/48 | 0/85 |  |
|  | p.Q61R | c.182A>G | 1/102 | 0/92 |  |
|  | p.Q61P | c.182A>C | 0/102 | 0/92 |  |
|  | p.Q61L | c.182A>T | 0/102 | 0/92 |  |
|  | p.Q61H | c.183A>C | 0/37 | 0/84 |  |
|  | p.Q61Q | c.183A>G | 0/37 | 0/84 |  |
|  | p.Q61H | c.183A>T | 0/37 | 0/84 |  |
|  | TOTAL CODON 12 |  | 0/78 | 0/82 |  |
|  | TOTAL CODON 13 |  | 0/89 | 1/92 |  |
|  | TOTAL CODON 61 |  | 0/37 | 0/84 |  |
|  | **completely succeeded ^#^** | | 0/30 | 1/75. |  |
|  | **percentage mutated ^##^** | | 0.94 (CI 0.02-5.14) | 1.08 (CI 0.03-5.85) | p=1, odds ratio 0.88, CI 0.023-34.09 |

Supplementary table S2A and S2B

| **GENE** | **AMINO ACID CHANGE** | **NUCLEOTIDE MUTATION** | **FLAT ADENOMAS** | **POLYPOID ADENOMAS** | **P-value^###^** |
| --- | --- | --- | --- | --- | --- |
| ***KRAS*** | p.G12C | c.34G>T | 2/105 | 7/93 |  |
|  | p.G12S | c.34G>A | 1/105 | 2/93 |  |
|  | p.G12R | c.34G>C | 0/105 | 0/93 |  |
|  | p.G12D | c.35G>A | 9/100 | 10/89 |  |
|  | p.G12V | c.35G>T | 5/100 | 9/89 |  |
|  | p.G12A | c.35G>C | 3/100 | 1/89 |  |
|  | p.G13D | c.38G>A | 7/105 | 4/93 |  |
|  | p.G13A | c.38G>C | 0/105 | 0/93 |  |
|  | p.G13V | c.38G>T | 0/105 | 0/93 |  |
|  | p.G13G | c.39C>A | 0/99 | 0/93 |  |
|  | p.G13G | c.39C>G | 0/99 | 0/93 |  |
|  | p.G13G | c.39C>T | 0/99 | 0/93 |  |
|  | p.A59T | c.175G>A | 0/89 | 0/88 |  |
|  | p.Q61K | c.181C>A | 0/102 | 0/93 |  |
|  | p.Q61E | c.181C>G | 0/102 | 0/93 |  |
|  | p.Q61L | c.182A>T | 1/105 | 0/93 |  |
|  | p.Q61R | c.182A>G | 0/105 | 0/93 |  |
|  | p.Q61P | c.182A>C | 0/105 | 0/93 |  |
|  | p.Q61H | c.183A>C | 0/84 | 0/89 |  |
|  | p.Q61H | c.183A>T | 0/84 | 0/89 |  |
|  | TOTAL CODON 12 |  | 20/99 | 29/89 | 0.05 |
|  | TOTAL CODON 13 |  | 7/98 | 4/93 |  |
|  | TOTAL CODON 61 |  | 1/81 | 0/89 |  |
|  | **completely succeeded ^#^** | | 20/66 | 32/82 |  |
|  | **percentage mutated ^##^** | | 27.85 (CI 19.58-37.39) | 36.3 (CI 26.57-46.92) | p=0.22, odds ratio 0.67 (CI 0.37-1.24)) |

Supplementary table S2C

| **GENE** | **AMINO ACID CHANGE** | **NUCLEOTIDE MUTATION** | **FLAT ADENOMAS** | **POLYPOID ADENOMAS** | **P-value^###^** |
| --- | --- | --- | --- | --- | --- |
| ***PIK3CA*** | p.G12D | c.35G>A | 0/74 | 0/86 |  |
|  | p.R38H | c.113G>A | 0/99 | 0/91 |  |
|  | p.E81K | c.241G>A | 0/103 | 0/92 |  |
|  | p.R88Q | c.263G>A | 0/104 | 0/92 |  |
|  | p.R93W | c.277C>T | 0/106 | 0/93 |  |
|  | p.G106V | c.317G>T | 0/97 | 0/91 |  |
|  | p.R108H | c.323G>A | 0/106 | 0/92 |  |
|  | p.G118D | c.353G>A | 0/106 | 0/93 |  |
|  | p.P134S | c.400C>T | 0/101 | 0/90 |  |
|  | p.S158L | c.473C>T | 0/105 | 0/93 |  |
|  | p.H160N | c.478C>A | 0/106 | 0/93 |  |
|  | p.K179T | c.536A>C | 0/71 | 0/77 |  |
|  | p.K184E | c.550A>G | 0/95 | 0/89 |  |
|  | p.N345K | c.1035T>A | 0/104 | 0/93 |  |
|  | p.C420R | c.1258T>C | 0/100 | 0/92 |  |
|  | p.P539R | c.1616C>G | 0/105 | 0/92 |  |
|  | p.E542K | c.1624G>A | 0/105 | 0/93 |  |
|  | p.E542Q | c.1624G>C | 0/105 | 0/93 |  |
|  | p.E545K | c.1633G>A | 0/105 | 0/93 |  |
|  | p.Q546K | c.1636C>A | 0/103 | 0/91 |  |
|  | p.Q546E | c.1636C>G | 0/103 | 0/91 |  |
|  | p.H701P | c.2102A>C | 0/105 | 0/90 |  |
|  | p.C901F | c.2702G>T | 0/104 | 0/92 |  |
|  | p.M1004I | c.3012G>T | 0/94 | 0/91 |  |
|  | p.G1007R | G3019C | 0/99 | 0/92 |  |
|  | p.H1047Y | c.3139C>T | 0/91 | 0/93 |  |
|  | p.H1047R | c.3140A>G | 0/104 | 0/92 |  |
|  | p.H1047L | c.3140A>T | 0/104 | 0/92 |  |
|  | p.G1049S | c.3145G>A | 0/106 | 0/92 |  |
|  | p.G1049R | c.3145G>C | 0/106 | 0/92 |  |
|  | exon 9 |  | 0/103 | 0/91 |  |
|  | exon 20 |  | 0/86 | 0/91 |  |
|  | **completely succeeded ^#^** | | 0/52 | 0/67 |  |
|  | **percentage mutated ^##^** | | 0 | 0 |  |

Supplementary table S2D

| **GENE** | **AMINO ACID CHANGE** | **NUCLEOTIDE MUTATION** | **FLAT ADENOMAS** | **POLYPOID ADENOMAS** | **P-value^###^** |
| --- | --- | --- | --- | --- | --- |
| ***PIK3R1*** | p.N564K | C/G | 0/84 | 0/89 |  |
|  | p.W583del | GGT/del | 0/82 | 0/85 |  |
|  | **completely succeeded ^#^** | | 0/80 | 0/85 |  |
|  | **percentage mutated ^##^** | | 0 | 0 |  |
|  |  |  |  |  |  |
| **GENE** | **AMINO ACID CHANGE** | **NUCLEOTIDE MUTATION** | **FLAT ADENOMAS** | **POLYPOID ADENOMAS** | **P-value^###^** |
| ***EGFR*** | p.E746_A750del | c.2235_2249del15 | 0/87 | 0/90 |  |
|  | p.E746_A750del | c.2236_2250del15 | 0/106 | 0/93 |  |
|  | **completely succeeded ^#^** | | 0/87 | 0/90 |  |
|  | **percentage mutated ^##^** | | 0 | 0 |  |
|  |  |  |  |  |  |
| **GENE** | **AMINO ACID CHANGE** | **NUCLEOTIDE MUTATION** | **FLAT ADENOMAS** | **POLYPOID ADENOMAS** | **P-value^###^** |
| ***PTEN*** | p.E150Q | c.448G>C | 0/106 | 0/93 |  |
|  | p.F241S | c.722T>C | 0/106 | 0/93 |  |
|  | p.K62R | c.185A>G | 0/94 | 0/86 |  |
|  | p.Y65C | c.194A>G | 0/96 | 0/91 |  |
|  | **completely succeeded ^#^** | | 0/87 | 0/85 |  |
|  | **percentage mutated ^##^** | | 0 | 0 |  |
|  |  |  |  |  |  |
| **GENE** | **AMINO ACID CHANGE** | **NUCLEOTIDE MUTATION** | **FLAT ADENOMAS** | **POLYPOID ADENOMAS** | **P-value^###^** |
| ***MAP2K4*** | p.Q142L | c.425A>T | 0/47 | 0/79 |  |
|  | p.R154W | c.460C>T | 0/74 | 0/91 |  |
|  | p.E221* | c.661G>T | 0/72 | 0/87 |  |
|  | p.S251N | c.752G>A | 0/82 | 0/91 |  |
|  | p.S280* | c.839C>A | 0/103 | 0/93 |  |
|  | p.I295fs*23 | c.882_882delG | 0/89 | 0/91 |  |
|  | p.K309N | c.927G>C | 0/49 | 0/85 |  |
|  | **completely succeeded ^#^** | | 0/36 | 0/76 |  |
|  | **percentage mutated ^##^** | | 0 | 0 |  |
|  |  |  |  |  |  |

Supplementary table S2E, S2F, S2G and S2H

| **GENE** | **AMINO ACID CHANGE** | **NUCLEOTIDE MUTATION** | **FLAT ADENOMAS** | **POLYPOID ADENOMAS** | **P-value^###^** |
| --- | --- | --- | --- | --- | --- |
| ***SMAD4*** | p.E330A | c.989A>C | 0/106 | 0/92 |  |
|  | p.D351N | c.1051G>A | 0/91 | 0/91 |  |
|  | p.D351H | c.1051G>C | 0/91 | 0/91 |  |
|  | p.G386R | c.1156G>C | 0/104 | 0/92 |  |
|  | p.A433V | c.1298C>T | 0/97 | 0/88 |  |
|  | p.R445* | c.1333C>T | 0/105 | 0/92 |  |
|  | p.Q245* | c.733C>T | 0/74 | 0/86 |  |
|  | **completely succeeded ^#^** | | 0/64 | 0/80 |  |
|  | **percentage mutated ^##^** | | 0 | 0 |  |
|  |  |  |  |  |  |
| **GENE** | **AMINO ACID CHANGE** | **NUCLEOTIDE MUTATION** | **FLAT ADENOMAS** | **POLYPOID ADENOMAS** | **P-value^###^** |
| ***FBXW7*** | p.R278* | c.832C>T | 0/61 | 1/90 |  |
|  | p.R465H | c.1394G>A | 1/102 | 0/88 |  |
|  | p.R479Q | c.1436G>A | 0/87 | 0/84 |  |
|  | p.V504I | c.1510G>A | 0/89 | 0/86 |  |
|  | p.R505C | c.1513C>T | 0/106 | 2/93 |  |
|  | p.R505H | c.1514G>A | 0/102 | 0/92 |  |
|  | **completely succeeded ^#^** | | 0/54 | 2/77 |  |
|  | **percentage mutated ^##^** | | 1.13 (CI 0.07-5.44) | 3.23 (CI 0.67-9.14) | p=0.41, odds ratio 0.35 CI 0.024-2.88 |

Supplementary table S2I and S2J

|  |  |  |  |  |  |
| --- | --- | --- | --- | --- | --- |
| **GENE** | **AMINO ACID CHANGE** | **NUCLEOTIDE MUTATION** | **FLAT ADENOMAS** | **POLYPOID ADENOMAS** | **P-value^###^** |
| ***CTNNB1*** | p.G34R | c.100G>A | 0/87 | 0/86 |  |
|  | p.S45F | c.134C>T | 1/106 | 1/93 |  |
|  | **completely succeeded ^#^** | | 1/87 | 0/85 |  |
|  | **percentage mutated ^##^** | | 0.94 (CI 0.02-5.14) | 1.08 (CI 0.03-5.85) | p=1, (odds ratio 0.88, CI 0.023-34.09) |
|  |  |  |  |  |  |
| **GENE** | **AMINO ACID CHANGE** | **NUCLEOTIDE MUTATION** | **FLAT ADENOMAS** | **POLYPOID ADENOMAS** | **P-value^###^** |
| ***STK11*** | p.Q170* | c.508C>T | 0/89 | 0/86 |  |
|  | **completely succeeded ^#^** | | 0/89 | 0/86 |  |
|  | **percentage mutated ^##^** | | 0 | 0 |  |
|  |  |  |  |  |  |

Supplementary table S2K and S2L

| **GENE** | **AMINO ACID CHANGE** | **NUCLEOTIDE MUTATION** | **FLAT ADENOMAS** | **POLYPOID ADENOMAS** | **P-value^###^** |
| --- | --- | --- | --- | --- | --- |
| ***PDGFRA*** | p.D1071N | c.3211G>A | 0/93 | 0/91 |  |
|  | p.T674I | c.2021C>T | 0/98 | 0/90 |  |
|  | p.V561D | c.1682T>A | 0/106 | 0/93 |  |
|  | **completely succeeded ^#^** | | 0/92 | 0/90 |  |
|  | **percentage mutated ^##^** | | 0 | 0 |  |
|  |  |  |  |  |  |

Supplementary table S2M

| **GENE** | **AMINO ACID CHANGE** | **NUCLEOTIDE MUTATION** | **FLAT ADENOMAS** | **POLYPOID ADENOMAS** | **P-value^###^** |  |
| --- | --- | --- | --- | --- | --- | --- |
| ***APC*** | p.Q1291* | c.3871C>T | 1/67 | 1/79 |  |  |
|  | p.T1301fs*15 | c.3900_3901insT | 0/67 | 1/79 |  |  |
|  | p.L1302fs*3 | c.3903delC | 0/67 | 3/79 |  |  |
|  | p.I1304fs*4 | c.3912delA | 0/67 | 1/79 |  |  |
|  | p.E1306* | c.3916G>T | 1/67 | 0/79 |  |  |
|  | p.E1309fs*4 | c.3921_3925delAAAAG | 1/74 | 2/79 |  |  |
|  | p.E1309* | c.3925G>T | 0/74 | 1/79 |  |  |
|  | p.K1310* | c.3928A>T | 1/74 | 0/79 |  |  |
|  | p.S1315* | c.3944C>A | 0/74 | 1/79 |  |  |
|  | *p.E1317Q* | *c.3949G>C* | 1/74 | 0/81 |  |  |
|  | p.V1320fs*11 | c.3957_3958insT | 0/74 | 1/81 |  |  |
|  | p.E1322* | c.3964G>T | 1/74 | 0/81 |  |  |
|  | p.R1331* | c.3991A>T | 0/74 | 1/81 |  |  |
|  | p.Q1338* | c.4012C>T | 0/74 | 1/81 |  |  |
|  | p.S1344* | c.4031C>A | 0/74 | 1/81 |  |  |
|  | p.E1353* | c.4057G>T | 0/74 | 1/81 |  |  |
|  | p.S1356* | c.4067C>G | 0/74 | 1/81 |  |  |
|  | p.Q1367* | c.4099C>T | 0/74 | 3/81 |  |  |
|  | p.Q1378fs*7 | c.4131_4132insT | 0/74 | 1/81 |  |  |
|  | p.Q1378* | c.4132C>T | 1/74 | 1/81 |  |  |
|  | p.E1397fs*1 | c.4184_4185insT | 0/74 | 1/81 |  |  |
|  | p.S1400* | c.4199C>A | 0/74 | 1/81 |  |  |
|  | p.Q1406fs*11 | c.4216_4217insCGTTC | 1/74 | 0/81 |  |  |
|  | p.Q1406* | c.4216C>T | 1/74 | 0/81 |  |  |
|  | p.E1408* | c.4222G>T | 1/74 | 0/81 |  |  |
|  | p.S1411fs*1 | c.4232_4238delGTGGAAT | 0/74 | 1/81 |  |  |
|  | p.S1411fs*4 | c.4233delT | 0/74 | 1/81 |  |  |
|  | p.V1414fs*5 | c.4241delT | 0/74 | 1/81 |  |  |
|  | p.G1416fs*3 | c.4247delG | 0/74 | 1/81 |  |  |
|  | p.P1420fs*2 | c.4259_4272delCCC  AGTGATCTTCC | 1/74 | 0/81 |  |  |
|  | p.S1421fs*52 | c.4263delT | 0/74 | 1/81 |  |  |
|  | p.Q1429* | c.4285C>T | 1/74 | 0/81 |  |  |
|  | p.T1430fs*43 | c.4287delC | 0/74 | 1/81 |  |  |
|  | p.P1432fs*35 | c.4294_4314delCACC  AAGCAGAAGTAAAAC | 1/74 | 0/81 |  |  |
|  | p.T1438fs*35 | c.4312delA | 0/74 | 1/81 |  |  |
|  | p.T1438fs*35 | c.4313delC | 0/74 | 1/81 |  |  |
|  | p.P1439fs*34 | c.4316delC | 1/74 | 0/81 |  |  |
|  | p.P1441fs*32 | c.4322delC | 0/74 | 1/81 |  |  |
|  | *p.P1442P* | *c.4326T>A* | 3/74 | 1/81 |  |  |
|  | p.T1445fs*28 | c.4333delA | 2/74 | 3/81 |  |  |
|  | p.T1445fs*28 | c.4334delC | 0/74 | 2/81 |  |  |
|  | p.R1450* | c.4348C>T | 4/74 | 2/81 |  |  |
|  | p.E1461* | c.4381G>T | 1/74 | 0/81 |  |  |
|  | p.E1461fs*7 | c.4382_4383insA | 1/74 | 0/81 |  |  |
|  | p.S1465fs*3 | c.4386_4387delGA | 0/60 | 3/81 |  |  |
|  | p.K1462fs*10 | c.4386delG | 0/60 | 1/81 |  |  |
|  | *p.P1483S* | *c.4447C>T* | 0/60 | 1/81 |  |  |
|  | *p.A1485T* | *c.4453G>A* | 1/60 | 0/81 |  |  |
|  | p.T1487fs*27 | c.4460_4464delCTTTA | 1/60 | 0/81 |  |  |
|  | p.L1488fs*19 | c.4464delA | 1/69 | 0/81 |  |  |
|  | p.H1490fs*17 | c.4469_4470insTA | 0/69 | 1/81 |  |  |
|  | p.A1492fs*22 | c.4473_4474insT | 1/69 | 0/81 |  |  |
|  | p.E1494fs*13 | c.4480delG | 0/69 | 1/81 |  |  |
|  | *p.G1499R* | *c.4495G>A* | 1/69 | 0/81 |  |  |
|  | **Completely succeeded** | | 30/60 | 47/79 |  |  |
|  | **percentage mutated ^##^** | | 30.27% (CI 20.13-42.05) | 48.49% (CI 37.23-59.87) | P=0.02 odds ratio 0.46 (CI 0.25-0.90) |  |
|  |  |  | three samples had a double mutation  (two missence and one truncating mutation) | one sample had a double mutation (missence mutation) |  |  |
|  |  | |  |  |  |  |

Supplementary table S2N
