# Supplementary material for: Comprehensive Mutation Analysis in Colorectal Flat Adenomas
Source: PLoS One. 2012 Jul 27;7(7):e41963. doi: 10.1371/journal.pone.0041963 (PMC3407043; doi:10.1371/journal.pone.0041963)
Supplement: Table S3 — Overview of all mutated samples. MSS; microsatelite stable, MSI; microsatelite instable, SSL; sessile serrated lesion, TSA; traditional serrated adenoma, Italic; silent mutation, #;Mutation not described before in colorectal adenomas, -; no data available. (DOC) [file pone.0041963.s003.doc]

| Sample | Type | Paris classification | Dysplasia | Size (mm) | Age | Gender | Location | Gene | Mutation | APC mutation |
| --- | --- | --- | --- | --- | --- | --- | --- | --- | --- | --- |
| 98 | Tubular | IIa | Moderate | 8 | 63 | M | Rectum | *KRAS* | p.G13D (c.38G>A) | p.Q1291* (c.3871C>T) |
| 107 | Tubulovillous | IIa | Moderate | 10 | 62 | M | Proximal | *KRAS* | p.G12D (c.35G>A) | *p.E1317Q (c.3949G>C)* |
| 89 | Tubular | IIa | Moderate | 10 | 66 | M | Proximal |  |  | p.E1322* (c.3964G>T) |
| 164 | Tubulovillous | IIa | Moderate | 8 | 60 | M | Distal |  |  | p.Q1406fs*11 (c.4216_4217insCGTTC) |
| 96 | Tubulovillous | IIa | Moderate | 5 | 75 | M | Proximal | *KRAS* | p.G12A (c.35G>C) | p.Q1406* (c.4216C>T) |
| 64 | Tubular | IIa | Moderate | 10 | 84 | M | Distal | *KRAS* | p.G12D (c.35G>A) | p.R1450* (c.4348C>T) |
| 65 | Tubulovillous | IIa | Moderate | 10 | 85 | F | Proximal | *KRAS* | p.G12V (c.35G>T) | p.R1450* (c.4348C>T) |
| 80 | Tubulovillous | IIa | Moderate | 10 | 75 | M | Proximal | *KRAS* | p.G13D (c.38G>A) | p.E1461fs*7 (c.4382_4383insA) |
| 117 | Tubular | IIa | Moderate | 8 | 65 | F | Distal |  |  | p.A1492fs*22 (c.4473_4474insT) |
| 116 | Tubulovillous | IIa | Moderate | 5 | 62 | M | Proximal | *KRAS* | p.G12C (c.34G>T) |  |
| 75 | Tubulovillous | IIa | Severe | 6 | 68 | M | Proximal | *KRAS* | p.G12D (c.35G>A) |  |
| 90 | Villous | IIa | Moderate | 10 | 79 | F | Proximal | *KRAS* | p.G12S (c.34G>A) |  |
| 134 | Tubular | IIa | Mild | 8 | 74 | M | Rectum | *KRAS* | p.Q61L (c.182A>T)^#^ |  |
| 59 | Tubular | IIa | Moderate | 5 | 71 | M | Proximal | *FBXW7* | p.R465H (c.1394G>A) |  |
| 119 | Tubulovillous | IIa | Moderate | 8 | 63 | F | Proximal | *KRAS* | p.G13D (c.38G>A) |  |
| 113 | Tubular | IIa | Moderate | 6 | 69 | F | Distal | *CTNNB1* | p.S45F (c.134C>T) |  |
| 88 | Tubular | IIa | Moderate | 10 | 75 | M | Distal |  | MSI |  |
| 94 | Tubular | IIc | Moderate | 6 | 58 | M | Proximal |  |  | p.Q1429* (c.4285C>T) |
| 383 | Tubular | Ip | Moderate | 9 | 69 | F | Rectum | *KRAS* | p.G13D (c.38G>A) | p.Q1291* (c.3871C>T) |
| 387 | Villous | Ip | Moderate | 9 | 65 | M | Rectum |  |  | p.T1301fs*15 (c.3900_3901insT) |
| 364 | Tubulovillous | Ip | Moderate | 10 | 57 | F | Rectum | *KRAS* | p.G12D (c.35G>A) | p.L1302fs*3 (c.3903delC) |
| 367 | Tubular | Ip | Moderate | 14 | 40 | F | Distal |  |  | p.L1302fs*3 (c.3903delC) |
| 361 | Tubulovillous | Ip | Moderate | 12 | 56 | F | Rectum |  |  | p.L1302fs*3 (c.3903delC) |
| 256 | Tubular | Ip | Moderate | 18 | 67 | F | Rectum |  |  | p.I1304fs*4 (c.3912delA) |
| 403 | Tubular | Ip | Moderate | 12 | 73 | M | Rectum |  |  | p.E1309fs*4 (c.3921_3925delAAAAG) |
| 389 | Tubular | Ip | Moderate | 10 | 70 | M | Distal |  |  | p.E1309fs*4 (c.3921_3925delAAAAG) |
| 253 | Tubulovillous | Ip | Moderate | 9 | 45 | F | Distal | *KRAS* | p.G12C (c.34G>T) | p.E1309* (c.3925G>T) |
| 262 | Tubular | Ip | Mild | 11 | 66 | F | Distal |  |  | p.S1315* (c.3944C>A) |
| 271 | Tubular | Ip | Mild | 6 | 62 | M | Proximal |  |  | p.V1320fs*11 (c.3957_3958insT) |
| 405 | Tubulovillous | Ip | Moderate | 10 | 66 | M | Rectum | *KRAS* | p.G12V (c.35G>T) | p.R1331* (c.3991A>T) |
| 408 | Tubular | Ip | Moderate | 7 | 76 | M | Rectum |  |  | p.Q1338* (c.4012C>T) |
| 360 | Tubular | Ip | Moderate | 15 | 77 | F | Distal |  |  | p.S1344* (c.4031C>A) |
| 386 | Tubulovillous | Ip | Severe | 10 | 52 | M | Rectum | *KRAS* | p.G12D (c.35G>A) | p.E1353* (c.4057G>T) |
| 368 | Tubular | Ip | Moderate | 13 | 60 | F | Rectum |  |  | p.S1356* (c.4067C>G) |
| 376 | Tubular | Ip | Moderate | 18 | 82 | F | Rectum | *FBXW7*  *KRAS* | p.R505C (c.1513C>T)^#^  p.G12D (c.35G>A) | p.Q1367* (c.4099C>T) |
| 274 | Tubular | Ip | Moderate | 7 | 54 | F | Distal |  |  | p.Q1367* (c.4099C>T) |
| 365 | Villous | Ip | Moderate | 6 | 69 | F | Rectum | *KRAS* | p.G12C (c.34G>T) | p.Q1367* (c.4099C>T) |
| 272 | Tubular | Ip | Moderate | 10 | 80 | F |  |  |  | p.Q1378fs*7 (c.4131_4132insT) |
| 411 | Tubular | Ip | Moderate | 17 | 74 | M | Distal |  |  | p.Q1378* (c.4132C>T)  *p.P1442P (c.4326T>A)* |
| 248 | Villous | Ip | Moderate | 60 | 70 | M | Distal | *FBXW7* | p.R278* (c.832C>T). | p.E1397fs*1 (c.4184_4185insT) |
| 375 | Tubular | Ip | Moderate | 10 | 54 | F | Distal |  |  | p.S1400* (c.4199C>A) |
| 257 | Tubular | Ip | Severe | 15 | 74 | M |  |  |  | p.S1411fs*1 (c.4232_4238delGTGGAAT) |
| 259 | Tubulovillous | Ip | Moderate | 20 | 75 | F | Distal |  |  | p.S1411fs*4 (c.4233delT) |
| 400 | Tubular | Ip | Moderate | 10 | 75 | M | Proximal | *KRAS* | p.G12C (c.34G>T) | p.V1414fs*5 (c.4241delT) |
| 380 | Villous | Ip | Moderate | 50 | 77 | F | Rectum | *KRAS* | p.G12D (c.35G>A) | p.G1416fs*3 (c.4247delG) |
| 401 | Tubular | Ip | Moderate | 15 | 56 | M | Distal |  |  | p.S1421fs*52 (c.4263delT) |
| 397 | Tubulovillous | Ip | Moderate | 20 | 57 | M | Proximal | *KRAS* | p.G12A (c.35G>C) | p.T1430fs*43 (c.4287delC) |
| 391 | Tubulovillous | Ip | Severe | 20 | 53 | M | Rectum | *FBXW7* | p.R505C (c.1513C>T)^#^ | p.T1438fs*35 (c.4312delA) |
| 288 | Tubular | Ip | Moderate | 9 | 77 | F | Distal | *KRAS* | p.G12C (c.34G>T) | p.T1438fs*35 (c.4313delC) |
| 402 | Tubular | Ip | Moderate | 9 | 79 | M | Rectum |  |  | p.P1441fs*32 (c.4322delC) |
| 392 | Tubular | Ip | Moderate | 18 | 81 | M | Rectum |  |  | p.T1445fs*28 (c.4333delA) |
| 393 | Tubulovillous | Ip | Moderate | 7 | 81 | M | Rectum |  |  | p.T1445fs*28 (c.4333delA) |
| 396 | Villous | Ip | Moderate | 20 | 81 | M | Rectum |  |  | p.T1445fs*28 (c.4333delA) |
| 378 | Villous | Ip | Moderate | 12 | 89 | F | Rectum | *KRAS* | p.G12V (c.35G>T) | p.T1445fs*28 (c.4334delC) |
| 385 | Tubulovillous | Ip | Moderate | 27 | 90 | F | Rectum | *KRAS* | p.G12V (c.35G>T) | p.T1445fs*28 (c.4334delC) |
| 413 | Tubulovillous | Ip | Moderate | 42 | 85 | M | Distal | *KRAS* | p.G13D (c.38G>A) | p.R1450* (c.4348C>T) |
| 398 | Tubular | Ip | Moderate | 50 | 60 | M | Rectum |  |  | p.R1450* (c.4348C>T) |
| 384 | Tubulovillous | Ip | Moderate | 25 | 85 | F | Proximal | *KRAS* | p.G12D (c.35G>A) | p.S1465fs*3 (c.4386_4387delGA) |
| 372 | Tubulovillous | Ip | Moderate | 21 | 66 | F | Rectum | *KRAS* | p.G12V (c.35G>T) | p.S1465fs*3 (c.4386_4387delGA) |
| 249 | Tubular | Ip | Mild | 8 | 74 | M | Proximal |  |  | p.S1465fs*3 (c.4386_4387delGA) |
| 270 | Tubulovillous | Ip | Moderate | 15 | 85 | F | Proximal |  |  | p.K1462fs*10 (c.4386delG) |
| 265 | Villous | Ip | Moderate | 10 | 68 | F | Rectum |  |  | *p.P1483S (c.4447C>T)* |
| 273 | Tubulovillous | Ip | Moderate | 40 | 40 | M | Rectum | *KRAS* | p.G12V (c.35G>T) | p.H1490fs*17 (c.4469_4470insTA) |
| 260 | Tubular | Ip | Moderate | 15 | 75 | F | Distal |  |  | p.E1494fs*13 (c.4480delG) |
| 374 | Tubulovillous | Ip | Moderate | 64 | 80 | F | Proximal | *CTNNB1*  *KRAS* | p.S45F (c.134C>T)  p.G12D (c.35G>A) |  |
| 245 | Tubulovillous | Ip | Moderate | 10 | 44 | F | Distal | *NRAS* | p.G13R (c.37G>C)^#^ |  |
| 251 | Tubulovillous | Ip | Moderate | 29 | 45 | F | Proximal | *KRAS* | p.G12C (c.34G>T) |  |
| 252 | Tubulovillous | Ip | Moderate | 30 | 45 | F | Proximal | *KRAS* | p.G12C (c.34G>T) |  |
| 258 | Tubular | Ip | Moderate | 20 | 61 | M | Proximal | *KRAS* | p.G12C (c.34G>T) |  |
| 269 | Tubular | Ip | Mild | 10 | 85 | F | Proximal | *KRAS* | p.G12S (c.34G>A) |  |
| 243 | Tubulovillous | Ip | Mild | 40 | 83 | F | Proximal | *KRAS* | p.G12D (c.35G>A) |  |
| 268 | Villous | Ip | Mild | 10 | 54 | M | Proximal | *KRAS* | p.G12D (c.35G>A) |  |
| 277 | Tubular | Ip | Moderate | 7 | 77 | M | Proximal | *KRAS* | p.G12D (c.35G>A) |  |
| 261 | Villous | Ip | Moderate | 10 | 69 | F | Rectum | *KRAS* | p.G12V (c.35G>T) |  |
| 404 | Tubulovillous | Ip | Severe | 20 | 81 | M | Rectum | *KRAS* | p.G12S (c.34G>A) |  |
| 370 | Tubular | Ip | Moderate | 10 | 81 | F | Distal | *KRAS* | p.G12D (c.35G>A) |  |
| 284 | Tubulovillous | Ip | Moderate | 20 | 77 | M | Proximal | *KRAS* | p.G12V (c.35G>T) |  |
| 266 | Tubulovillous | Ip | Moderate | 16 | 66 | F |  | *KRAS* | p.G13D (c.38G>A) |  |
| 369 | Tubular | Ip | Severe | 37 | 64 | F | Rectum | *KRAS* | p.G13D (c.38G>A) |  |
| 246 | Tubular | Ip | Severe | 10 | 68 | F | Distal | *BRAF* | p.V600E (c.1799T>A) |  |
| 286 | Tubulovillous | Ip | Moderate | 9 | 87 | F | Proximal | *BRAF* | p.V600E (c.1799T>A) |  |
| 394 | Tubulovillous | Ip | Moderate | 12 | 68 | M | Distal | *KRAS* | p.G12V (c.35G>T) |  |
| 362 | Tubular | Ip | Moderate | 10 | 61 | F | Rectum | *KRAS* | p.G12V (c.35G>T) |  |
| 85 | Tubulovillous | LST-F | Moderate | 30 | 85 | F | Rectum |  |  | p.E1306* (c.3916G>T) |
| 92 | Tubulovillous | LST-F | Moderate | 30 | 73 | M | Rectum | *NRAS* | p.Q61R (c.182A>G) ^#^ | p.E1309fs*4 (c.3921_3925delAAAAG) |
| 73 | Tubular | LST-F | Moderate | 20 | 82 | M | Proximal |  |  | p.K1310* (c.3928A>T) |
| 102 | Tubular | LST-F | Moderate | 11 | 75 | M | Rectum |  |  | p.Q1378* (c.4132C>T) |
| 74 | Tubulovillous | LST-F | Moderate | 20 | 60 | M | Proximal |  |  | p.E1408* (c.4222G>T)  *p.P1442P (c.4326T>A)* |
| 67 | Tubular | LST-F | Severe | 25 | 79 | F | Proximal |  |  | p.P1420fs*2 (c.4259_4272delCCCAGTGAT  CTTCC) p.R1450* (c.4348C>T) |
| 69 | Tubular | LST-F | Mild | 12 | 54 | F | Proximal | *KRAS* | p.G13D (c.38G>A) | p.P1432fs*35 (c.4294_4314delCACCAAGC  AGAAGTAAAAC) |
| 95 | Tubular | LST-F | Moderate | 12 | 61 | M | Rectum |  |  | p.P1439fs*34 (c.4316delC) |
| 166 | Tubulovillous | LST-F | Moderate | 12 | 65 | F | Proximal |  |  | *p.P1442P (c.4326T>A)* |
| 109 | Tubulovillous | LST-F | Moderate | 25 | 83 | F | Proximal | *KRAS* | p.G12D (c.35G>A) | p.T1445fs*28 (c.4333delA) |
| 87 | Tubular | LST-F | Mild | 20 | 59 | M | Proximal |  |  | p.E1461* (c.4381G>T) |
| 111 | Tubulovillous | LST-F | Moderate | 35 | 78 | M | Proximal |  |  | p.T1487fs*27 (c.4460_4464delCTTTA) |
| 61 | Tubular | LST-F | Severe | 15 | 47 | M | Proximal |  |  | *p.G1499R (c.4495G>A)* |
| 70 | Tubulovillous | LST-F | Moderate | 20 | 86 | F | Proximal | *KRAS* | p.G12A (c.35G>C) |  |
| 154 | Tubulovillous | LST-F | Moderate | 30 | 85 | M | Proximal | *KRAS* | p.G12A (c.35G>C) |  |
| 82 | Tubulovillous | LST-F | Moderate | 30 | 80 | F | Proximal | *KRAS* | p.G12V (c.35G>T) |  |
| 167 | Tubulovillous | LST-F | Moderate | 20 | 69 | M | Proximal | *KRAS* | p.G12V (c.35G>T) |  |
| 79 | Tubulovillous | LST-F | Moderate | 35 | 84 | F | Proximal | *KRAS* | p.G13D (c.38G>A) |  |
| 114 | Tubular | LST-F | Severe | 20 | 57 | M | Proximal | *KRAS* | p.G13D (c.38G>A) |  |
| 63 | Serrated (TSA) | LST-F | Severe | 50 | 84 | M | Proximal | *BRAF* | p.V600E (c.1799T>A) |  |
| 81 | Serrated (SSL) | LST-F | Moderate | 17 | 65 | M | Proximal | *BRAF* | p.V600E (c.1799T>A) |  |
| 78 | Serrated (SSL) | LST-F | Moderate | 20 | 82 | F | Proximal | *BRAF* | p.V600E (c.1799T>A) |  |
| 68 |  | LST-F | Moderate | 100 | 87 | F | Rectum | *KRAS* | p.G12D (c.35G>A) |  |
| 124 | Tubular | LST-F | Moderate | 20 | 75 | M | Proximal | *KRAS* | p.G12D (c.35G>A) |  |
| 148 | Tubulovillous | LST-G | Moderate | 25 | 69 | F | Proximal | *KRAS* | p.G12V (c.35G>T) | p.T1445fs*28 (c.4333delA) |
| 168 | Tubular | LST-G | Moderate | 30 | 64 | F | Proximal |  |  | p.R1450* (c.4348C>T)  *p.P1442P (c.4326T>A)* |
| 173 | Tubular | LST-G | Moderate | 11 | 72 | M | Rectum |  |  | *p.A1485T (c.4453G>A)* |
| 151 | Tubular | LST-G | Moderate | 12 | 61 | M | Rectum | *KRAS* | p.G13D (c.38G>A) | p.L1488fs*19 (c.4464delA) |
| 58 | Tubular | LST-G | Moderate | 30 | 82 | F | Rectum | *KRAS* | p.G12D (c.35G>A) |  |
| 120 | Tubular | LST-G | Severe | 20 | 66 | F | Rectum | *KRAS* | p.G12C (c.34G>T) |  |
| 160 | Tubular | LST-G | Moderate | 16 | 76 | M | Proximal | *KRAS* | p.G12D (c.35G>A) |  |
| 170 | Tubular | LST-G | Moderate | 15 | 70 | M | Distal | *KRAS* | p.G12V (c.35G>T) |  |
| 150 | Tubular | LST-G | Moderate | 20 | 53 | F | Proximal | *KRAS* | p.G12D (c.35G>A) |  |

Supplementary table S3
